# Phylogenetic Revision of Savoryellaceae and Evidence for Its Ranking as a Subclass

**DOI:** 10.3389/fmicb.2019.00840

**Published:** 2019-05-07

**Authors:** Monika C. Dayarathne, Sajeewa S. N. Maharachchikumbura, E. B. Gareth Jones, Wei Dong, Bandarupalli Devadatha, Jing Yang, Anusha H. Ekanayaka, Wasana De Silva, Vemuri V. Sarma, Abdullah M. Al-Sadi, Kitiphong Khongphinitbunjong, Kevin D. Hyde, Rui Lin Zhao

**Affiliations:** ^1^Center of Excellence in Fungal Research, Mae Fah Luang University, Chiang Rai, Thailand; ^2^World Agroforestry Centre East and Central Asia Office, Kunming, China; ^3^Key Laboratory for Plant Biodiversity and Biogeography of East Asia, Kunming Institute of Botany, Chinese Academy of Sciences, Kunming, China; ^4^Department of Botany and Microbiology, College of Sciences, King Saud University, Riyadh, Saudi Arabia; ^5^Department of Crop Sciences, College of Agricultural and Marine Sciences, Sultan Qaboos University, Muscat, Oman; ^6^Department of Biotechnology, School of Life Sciences, Pondicherry University, Kalapet, India; ^7^Donghu Experimental Station of Lake Ecosystems, State Key Laboratory of Freshwater Ecology and Biotechnology, Institute of Hydrobiology, Chinese Academy of Sciences, Wuhan, China; ^8^School of Science, Mae Fah Luang University, Chiang Rai, Thailand; ^9^State Key Laboratory of Mycology, Institute of Microbiology, Chinese Academy of Sciences, Beijing, China; ^10^College of Life Sciences, University of Chinese Academy of Sciences, Beijing, China

**Keywords:** freshwater, marine, morphology, phylogeny, Savoryellomycetidae, taxonomy

## Abstract

Morphology, phylogeny, and molecular clock analyses were carried out on Savoryellaceae in order to understand the placements of taxa in this family. *Ascotaiwania* and *Neoascotaiwania* formed a well-supported separate clade in the phylogeny of concatenated partial SSU, LSU, TEF, and RPB2 gene data. These two genera share similar morphological features, especially in their asexual morphs, indicating that they are congeneric. Hence, we synonymize *Neoascotaiwania* under *Ascotaiwania*. *Ascotaiwania hughesii* (and its asexual morph, *Helicoon farinosum*) and *Monotosporella setosa* grouped in a clade sister to Pleurotheciales and are excluded from *Ascotaiwania* which becomes monophyletic. A novel genus *Helicoascotaiwania* is introduced to accommodate *Ascotaiwania hughesii* and its asexual morph, *Helicoon farinosum*. A novel species, *Savoryella yunnanensis* is introduced from a freshwater habitat in Yunnan Province, China. Comprehensive descriptions and illustrations are provided for selected taxa in this family. In addition, we provide evolutionary divergence estimates for Savoryellomycetidae taxa and major marine based taxa to support our phylogenetic and morphological investigations. The taxonomic placement of these marine-based taxa is briefly discussed. Our results indicate that the most basal group of marine-based taxa are represented within Lulworthiales, which diverged from ancestral Sordariomycetes around 149 Mya (91–209) and Savoryellomycetidae around 213 Mya (198–303).

## Introduction

The family Savoryellaceae (Savoryellales) was established by Jaklitsch and Réblová (2015) and is typified by the genus *Savoryella*. Boonyuen et al. (2011) had earlier introduced the order Savoryellales, but without designating a family. According to phylogenetic and molecular clock analyses (Hongsanan et al., 2017; Hyde et al., 2017), the orders Conioscyphales, Fuscosporellales, Pleurotheciales, and Savoryellales cluster together as a distinct clade, with a stem age of 268 Mya. Hence, the order Savoryellales was referred to a new subclass Savoryellomycetidae by Hongsanan et al. (2017) and is supported in the present study. The genus *Savoryella*, based on morphological features, was previously placed in the *Sordariales* genera *incertae sedis* by Jones et al. (2009) and, later, Boonyuen et al. (2011) showed that *Savoryella, Ascotaiwania, Ascothailandia*, and *Canalisporium* cluster in the order *Savoryellales* within Hypocreomycetidae, Sordariomycetes. According to the one fungus-one name concept (Hawksworth, 2011), the genus *Canalisporium* was recommended for protection over *Ascothailandia* hence, it was synonymized under *Canalisporium* based on sequence data (Sri-indrasutdhi et al., 2010). Maharachchikumbura et al. (2016) and Wijayawardene et al. (2018) accepted the placement of Savoryellaceae in the order *Savoryellales.* With the recent inclusion of the genus *Neoascotaiwania*, Savoryellaceae comprised four genera: *Ascotaiwania, Canalisporium*, and *Savoryella* (Hernández-Restrepo et al., 2017). Taxa presently referred to this family total 40 species (Table 1).

**Table 1:** Genera referred to Savoryellaceae (before our family treatment).

Genus	No. of accepted species	No. of species with molecular data
*Ascotaiwania*	12	7
*Canalisporium*^∗^	15	10
*Neoascotaiwania*	2	2
*Savoryella*	11	6

*^∗^Only certain species are included within Savoryellaceae.*

### Distribution and Occurrence of Savoryellaceae Species

**FIGURE 1:**
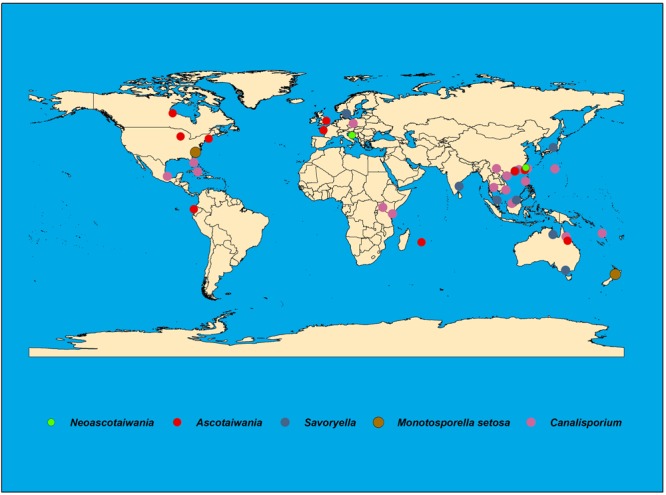
Worldwide distribution of Savoryellaceae species.

Species of Savoryellaceae are abundant in submerged wood in aquatic habitats, viz fresh, saline or brackish water and some species have been reported from terrestrial woody plants (Linder, 1929; Jones et al., 2015). *Ascotaiwania* species are isolated from submerged and decaying wood in freshwater habitats (Sivanesan and Chang, 1992; Ranghoo et al., 1999), and are widely distributed in countries such as Ecuador, France, Great Britain, Hong Kong, Malaysia, Mauritius, Taiwan, Thailand, and Australia (Figure 1). *Canalisporium* species are saprobes mostly on rotten wood and distributed in tropical regions of both hemispheres (Figure 1) (Goh et al., 1998). Further, they occur on woody plants (Rao and de Hoog, 1986; Raja and Shearer, 2007) and submerged, decaying wood in freshwater (Sivichai et al., 1998; Tsui et al., 2001). *Canalisporium* species have been recorded from Cuba, India, Kenya, Malaysia, Taiwan, Thailand, and Uganda (Holubová-Jechová and Sierra, 1984; Kirk, 1985; Rao and de Hoog, 1986; Matsushima, 1987; Nawawi and Kuthubutheen, 1989; Sri-indrasutdhi et al., 2010). Though *Savoryella* species are cosmopolitan in distribution, mostly they are common in tropical and subtropical ecosystems (Jones et al., 2016) (Figure 1). *Neoascotaiwania* species have been documented from forest soil in Spain and decaying wood collected in streams in Taiwan (Chang et al., 1998; Hernández-Restrepo et al., 2017) (Figure 1 and Table 2).

**Table 2:** Different hosts/substrates of Savoryellaceae species (these species consideration before the family treatment in this study).

Species	Host/substrate	Reference
***Ascotaiwania *fusiformis****	On dead decaying submerged wood	Yang et al., 2016
***Ascotaiwania *hsilio****^F^	Decaying wood submerged in a stream	Chang et al., 1998
***Ascotaiwania *hughesii****	On decorticated wood submerged in lake	Fallah et al., 1999
***Ascotaiwania *licualae****^F^	Dead petiole of *Licuala ramsayi*	Fröhlich and Hyde, 2000
***Ascotaiwania *lignicola****^F^	Deadwood	Chang et al., 1998
***Ascotaiwania *mauritiana****^F^	Prop root of *Pandanus palustris*	Dulymamode et al., 2001
***Ascotaiwania *mitriformis****^F^	Submerged wood	Ranghoo and Hyde, 1998
***Ascotaiwania *pallida****^F^	Wood submerged in a river	Hyde and Goh, 1999
***Ascotaiwania *palmicola****^F^	Dead *Iriartea* rachis	Hyde, 1995
***Ascotaiwania *pennisetorum****^F^	On standing senescent culms of *Pennisetum purpureum*	Wong and Hyde, 2001
***Ascotaiwania *sawadae****^F^	Wood submerged in a stream	Chang et al., 1998
***Ascotaiwania *wulai****^F^	Decaying twigs submerged in a stream	Chang et al., 1998
***Canalisporium *caribense****^T,F^	*Areca catechu, Arundinaria alpine, Arundinaria* sp., *Bambusa* sp., *Amomum siamense, Archontophoenix alexandrae, Dipterocarpus* sp., *Drymophloeus pachycladus, Freycinetia multiploa, Freycinetia* sp., *Juncus conglomeratus, Licuala longicalycata, Musa acuminate, Pandanus simplex, Pandanus* sp., *Pandanus tectorius, Rorippa amphibian, Syzygium jambos*, palm	Bussaban et al., 2001; Zhang et al., 2014
***Canalisporium dehongense***^F^	Submerged wood in a small river	Hyde et al., unpublished
***Canalisporium elegans***^T^	Rotten branch	Goh et al., 1998; Zhang et al., 2014
***Canalisporium exiguum***^F^	Submerged wood	Goh et al., 1998
***Canalisporium grenadoideum***^F^	Deadwood of *Wrightia tomentosa* submerged in a stream	Sri-indrasutdhi et al., 2010
***Canalisporium jinghongensis***^F^	Submerged wood	Zhang et al., 2014
***Canalisporium kenyense***^T^	Rotten wood	Goh et al., 1998
***Canalisporium krabiense***^T^	Leaf sheath of *Pandanus* sp.	Tibpromma et al., 2018
***Canalisporium microsporum***^T^	Rotten wood	Zhao et al., 2012
***Canalisporium nigrum***^T^	The rotten sheath of palm:	Zhang et al., 2014
***Canalisporium pallidum***^F^	Submerged wood: Hong Kong	Zhang et al., 2014
***Canalisporium panamense***^F^	Decorticated wood submerged in river	Ferrer and Shearer, 2005
***Canalisporium pulchrum***^T^	Rotten branch	Zhang et al., 2014
***Canalisporium thailandensis***^T^	Dead leaf sheath of *Pandanus* sp.	Tibpromma et al., 2018
***Canalisporium variabile***^F^	Wood submerged in a creek	Goh and Hyde, 1999
***Neoascotaiwania limnetica***^F^	Decaying wood collected in a stream	Chang et al., 1998
***Neoascotaiwania terrestris***^T^	Forest soil	Hernández-Restrepo et al., 2017
***Savoryella appendiculata***^F,M^	Driftwood, marine	Boonyuen et al., 2011
***Savoryella aquatica***^F^	*Machilus* sp., *Machilus velutina*, freshwater submerged wood	Hyde, 1993
***Savoryella curvispora***^F^	Submerged branch	Ho et al., 1997
***Savoryella fusiformis***^F^	*Machilus* sp., *Machilus velutina, Pinus massoniana, Pinus* sp.	Ho et al., 1997
***Savoryella grandispora***^F,M^	Submerged wood	Hyde, 1994
***Savoryella lignicola***^F,M^	*Bambusa* sp., *Machilus* sp., *Machilus velutina, Phragmites australis, Pinus* sp., *Pinus massoniana, Rhizophora mucronata*, wood, submerged	Hyde, 1993
***Savoryella longispora***^M^	Intertidal mangrove wood	Hyde, 1993
***Savoryella melanospora***^M^	Driftwood partially buried in the sand	Abdel-Wahab and Jones, 2000
***Savoryella paucispora***^F,M^	*Avicennia marina, Pinus* sp.	Manimohan et al., 2011
***Savoryella verrucosa***^F^	Wood	Ho et al., 1997

*F, freshwater; M, marine; T, terrestrial.*

### Morphological Significance

Members of Savoryellaceae have perithecial, flask-shaped ascomata with a periphysate ostiole, which is central or eccentric when horizontally lying on the host, papillate or with a short/long neck, conical or cylindrical (Jones and Eaton, 1969; Kohlmeyer and Kohlmeyer, 1979; Eriksson and Hawksworth, 1987; Barr, 1990; Read et al., 1993; Jones and Hyde, 1992; Tsui and Hyde, 2003; Réblová et al., 2016a). Asci of Savoryellaceae are clavate to cylindrical, persistent, with an inamyloid apical ring (Kohlmeyer, 1986; Eriksson and Hawksworth, 1986, 1987; Read et al., 1993; Jones and Hyde, 1992; Tsui and Hyde, 2003; Jones et al., 2009; Boonyuen et al., 2011). Ascomata of *Savoryella* are long-necked and pale in color while, ascomata of *Ascotaiwania* are dark-brown to black with a short or long, lateral neck (Chang et al., 1998; Tsui and Hyde, 2003). Ascomatal morphology of *Neoascotaiwania* also mostly similar to that of *Ascotaiwania* (Hernández-Restrepo et al., 2017). Further, the sexual morph of *Canalisporium* (=*Ascothailandia*), comprises globose, dark brown, ostiolate ascomata (Sri-indrasutdhi et al., 2010). In the protolog of *Savoryella*, the apical pore or apparatus of ascus was not described (Jones and Eaton, 1969). Later, Jones and Hyde (1992) observed the asci and apical apparatus of *Savoryella appendiculata, S. longispora*, and *S. paucispora*. Ultrastructural observations of asci and ascospores with transmission electron microscopy (TEM) by Read et al. (1992) described the ascal apical ring of *S. appendiculata* and *S. longispora* as extending subapically on to the side walls of the ascus and similar to that described for *Aniptodera chesapeakensis*, which comprises retraction of the plasma membrane at the apex. The ascal apical ring of *Ascotaiwania* is comparatively large and highly developed, while *Canalisporium* (=*Ascothailandia*) has a J-prominent apical ring (Sri-indrasutdhi et al., 2010; Zhang et al., 2014). Paraphyses of *Savoryella* are inconspicuous at maturity while, *Ascotaiwania* has thin, filiform, septate, up to 2 μm wide paraphyses which deliquesce early and are rarely seen in mature ascomata (Chang et al., 1998; Tsui and Hyde, 2003; Boonyuen et al., 2011). Asci of *Ascotaiwania* are cylindrical, unitunicate with a prominent J-refractive and well-developed apical apparatus comprises an electron-dense apical ring with a plug. The plug deliquesces prior to ascospore release (Chang et al., 1998; Boonyuen et al., 2011). Asci of *Neoascotaiwania* are only differed from *Ascotaiwania* by having a thinner apical ring (Hernández-Restrepo et al., 2017). Ultrastructural studies of *S. lignicola* revealed that the unitunicate ascus wall comprised an outer, 30–40 nm electron-dense layer and an inner, 420–450 nm, thick, electron-transparent layer (Read et al., 1993).

*Savoryella* ascospores are ellipsoid to fusiform, with several septa, and versicolorous with brown median cells and hyaline polar cells. The ascospores of *Savoryella appendiculata* and *S. paucispora* have mucilaginous sheath around the central cells (Boonyuen et al., 2011). *Savoryella appendiculata* is the solitary species in this genus with ascospores with polar tetradiate appendages, formed as an outgrowth of the hyaline apical cell of the ascospore on release from the ascus (Jones and Hyde, 1992; Read et al., 1993). *Ascotaiwania* ascospores often have three or more than three septa, and are versicolored (Chang et al., 1998; Hernández-Restrepo et al., 2017) in which ascospores are surrounded by a thin mucilaginous sheath and an ascospore wall comprising an electron-dense episporium and a less electron-dense mesosporium (Read et al., 1993). Further, external to the episporium is a fibro-granular sheath (Read et al., 1993). Ascospores of *Ascothailandia* are also fusiform, straight or curved, three euseptate and versicolored (Sri-indrasutdhi et al., 2010). Considering the morphologies of asexual morphs of Savoryellaceae; conidia of *Canalisporium* species are muriform, flattened dorsoventrally, with a layer of punctually arranged cells, that are aided by a small, thin-walled, cuneiform, pale basal cell (Goh et al., 1998). Conidia possess a single column of vertical septa, and evenly spaced several rows of transverse septa (Boonyuen et al., 2011). The most remarkable feature of the conidia is the presence of narrow canals connecting adjacent cell lumens and surrounded by a highly pigmented ring, observed as a circular disc in lateral view or a barrel shape in dorsiventral view (Moore, 1959; Ellis, 1971, 1976; Ho et al., 2002). Asexual morphs of *Ascotaiwania* are bactrodesmium-like, monotosporella-like, monodictys-like or trichocladium-like (Ranghoo and Hyde, 1998; Chang, 2001; Tsui and Hyde, 2003; Réblová et al., 2016a). Species in the genera *Ascotaiwania, Savoryella*, and *Canalisporium* have common phenotypic characteristics, such as similar ascomata, paraphyses and versicolored ascospores (Boonyuen et al., 2011).

### Key to Genera of Savoryellaceae

1. Sexual morph perithecial………………………………**2**
1. Asexual morph hyphomycetous…………***Canalisporium***
2. Asci comprises massive, refractive, apical ring and ascospores fusiform, with pointed end cells…***Ascotaiwania***
2. Asci comprises small, discoid, apical pore/ring and ascospores ellipsoid, with rounded end cells….. ***Savoryella***

Note: Key to genera only for the accepted genera in this study.

## Materials and Methods

### Examination of Specimen, Fungal Sampling, and Morphology

Herbarium specimens were loaned from the herbaria: BRIP and IMI^1^. Fresh material was collected on submerged decaying wood of *Avicennia marina* in Tamil Nadu, India and submerged wood in a small river in Yunnan, China. Specimens were brought to the laboratory in plastic bags and incubated in plastic boxes at 25°C. The samples were processed, examined, and photographed following the method described in Dayarathne et al. (unpublished).

### Taxon Sampling, Molecular Data, and Phylogenetic Analysis

A representative species of 152 of Sordariomycetes (including 24 strains which represent 20 species belonging to the family Savoryellaceae) were selected and *Leotia lubrica* was selected as the outgroup taxon (Supplementary Table 1) following Samarakoon et al. (2016). Sequence data were obtained from GenBank based on previous literature (Supplementary Table 1) (Samarakoon et al., 2016; Hongsanan et al., 2017). Multiple sequence alignments were generated from four loci (LSU, SSU, RPB2, and TEF1-α) following Hall (1999) and Dayarathne et al. (unpublished). The final alignment consisted of 4,637 characters (LSU = 1,291 bp, SSU = 1,091 bp, RPB2 = 1,092 bp, and TEF1-α = 1,048 bp) and gaps were treated as missing data. Nucleotide substitution models were determined with MrModeltest v. 2.2 (Nylander, 2004). The GTR+I+G model was used in the analysis for phylogenetic and divergence time estimation studies.

Phylogenetic analyses of the sequence data consisted of maximum likelihood (ML) and Bayesian inference analyses (BI). The ML trees were generated following Stamatakis et al. (2008), Miller et al. (2010), and Dayarathne et al. (unpublished). The Bayesian tree was obtained by using MCMC sampling in MrBayes v3.1.2 (Huelsenbeck and Ronquist, 2001; Zhaxybayeva and Gogarten, 2002) using the parameters described by Dayarathne et al. (unpublished). The resulting trees were printed with FigTree v.1.4.0 (Rambaut, 2012) and the final layout was done with Microsoft PowerPoint.

### Molecular Clock Analysis

Bayesian analysis was conducted for the estimation of divergence times using molecular data of multi-gene loci and incorporating fossil data and secondary calibration estimations. BEAST v1.8.0 software was used to obtained divergence time estimates were performed using the BEAUti (BEAST package) was aided to prepare XML file including the partitioned alignment. The analysis were generally similar to our previous work of Samarakoon et al. (2016), and the details are as follows. The GTR+I+G was used as models of substitution for each gene regions. Tree priors were set and maximum clade credibility (MCC) trees were obtained as described by Samarakoon et al. (2016). Trees were visualized using FigTree^2^ and the final layout was optimized with Adobe Illustrator.

### Fossil Calibration

A fossil belongs to the subclass Hypocreomycetidae order Hypocreales, *Paleoophiocordyceps coccophagus* was used in calibrating the tree (Samarakoon et al., 2016). A fossilized male scale for insect (Albicoccidae) parasitized by *P. coccophagus* from the late Mesozoic (Upper Albian) period in Burmese amber was investigated by Sung et al. (2007) and Sadowski et al. (2016) which is the oldest fossil record as evidence for the fungal-animal parasitism. Morphological characters of the fossil *P. coccophagus* were reminiscent of asexual morph characteristics of *Hirsutella* and *Hymenostilbe* (Sung et al., 2007) that are synonyms of *Ophiocordyceps* (Ophiocordycipitaceae, Hypocreales; Quandt et al., 2014). Fossil age of *P. coccophagus* has been estimated as around 99–105 Mya according to the geological timescale (Cruickshank and Ko, 2003). Albian period is estimated as 100–113 Mya according to the geological timescale of Walker et al. (2012). Above fossil data was used to calibrate the crown node of the genus *Ophiocordyceps* (exponential distribution, offset 100, mean 27.5, with 97.5% credibility interval of 201.4 Mya).

### Secondary Calibration

Secondary calibration determination was done according to Samarakoon et al. (2016).

## Results

### Phylogenetic Analysis

The ML analyses of concatenated sequence alignment with LSU, SSU, RPB2, and TEF1-α dataset generated best scoring RAxML tree (Figure 1) with a final ML optimization likelihood value of -14416.427031. Different parameters for ML analyses are summarized in Supplementary Table 2. For concatenated LSU, SSU, RPB2, and TEF1-α dataset, six simultaneous Markov chains were run for 1.5 M generations and trees were sampled every 100th generation resulting in 15,001 total trees. Of these 13,501 trees were used to calculate the posterior probabilities (PP) in the majority rule consensus tree, after discarding the first 1,500 trees that representing the burn-in phase (10%) of the analysis. Overall, tree topologies obtained from of the ML and BI analyses were similar to each other.

**FIGURE 2:**
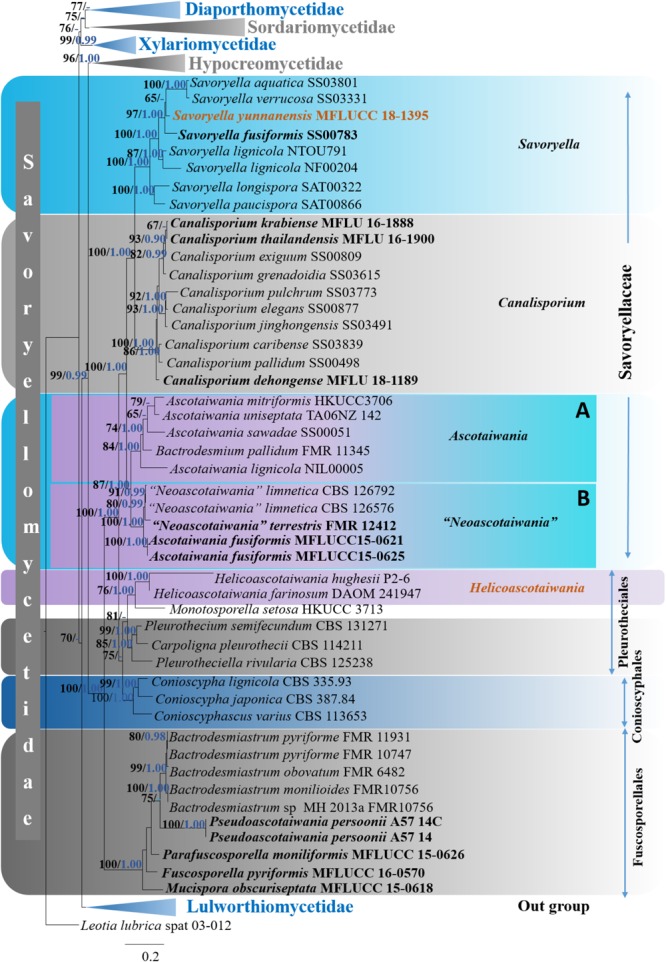
Phylogram generated from maximum likelihood analysis based on combined LSU, SSU, RPB2, and TEF1-α sequence data of selected taxa from Sordariomycetes. One hundred and fifty-two strains are included in the analyses. Single gene analyses were carried out and compared with each species, to compare the topology of the tree and clade stability. The tree was rooted with *Leotia lubrica* (spat 03-012). Bayesian posterior probabilities (PP, blue) >0.90 and maximum likelihood bootstrap (ML, black) values >60% are given above the nodes. The scale bar indicates 0.2 changes and new isolate is in blue.

Six distinct clades (Diaporthomycetidae, Hypocreomycetidae, Lulworthiomycetidae, Sordariomycetidae, Savoryellomycetidae, and Xylariomycetidae) were recognized within the phylogenetic tree. Subclass Savoryellomycetidae formed a robust clade (100% ML, 1.00 PP, Figure 2) within the class Sordariomycetes. Five subclades comprising Conioscyphaceae, Fuscosporellaceae, Pleurotheciaceae, Savoryellaceae, and a clade with *Ascotaiwania hughesii* (DAOM 241947, P2-6) strains and *Monotosporella setosa* (HKUCC 3713) were recognized within the subclass Savoryellomycetidae.

The genera *Ascotaiwania*, “*Neoascotaiwania*,” *Canalisporium* and *Savoryella* formed well-separated subgroups within the family Savoryellaceae that is monophyletic.

***Ascotaiwania, Neoascotaiwania* clade**: *Ascotaiwania, Neoascotaiwania*, and *Bactrodesmium pallidum* (FMR 11345) collectively formed a robust clade (100% ML, 1.00 PP, Figure 2) within Savoryellaceae, which splits into two subclades (A & B). Sub clade A comprised *Neoascotaiwania limnetica* (CBS 126576, CBS 126792), *Neoascotaiwania terrestris* (CBS 142291), and *Ascotaiwania fusiformis* (MFLUCC15 0621, MFLUCC15 0625) (Figure 2), while *Ascotaiwania mitriformis* (HKUCC3706), *Ascotaiwania sawadae* (SS00051), *Bactrodesmium pallidum* (FMR 11345), *Ascotaiwania lignicola* (NIL00005) grouped in subclade B. ***Canalisporium* clade**: *Canalisporium caribense* (SS03839), *Canalisporium elegans* (SS00877), *Canalisporium exiguum* (SS00809), *Canalisporium grenadoidia* (SS03615), *Canalisporium jinghongensis* (SS03491), *Canalisporium pallidum* (SS00498) and *Canalisporium pulchrum* (SS03773) formed a monophyletic clade with strong support (100% ML, 1.00 PP, Figure 2) within Savoryellaceae. ***Savoryella* clade**: A newly generated sequence of *Savoryella yunnanensis* (MFLUCC 18-1395) grouped with *Savoryella aquatica* (SS03801), *Savoryella fusiformis* (SS00783), *Savoryella lignicola* (NF00204, NTOU791), *Savoryella longispora* (SAT00322), *Savoryella paucispora* (SAT00866) and *Savoryella verrucosa* (SS03331). ***Ascotaiwania hughesii* clade**: *Ascotaiwania hughesii* (P2-6, DAOM 241947 “*Helicoon farinosum*”) and *Monotosporella setosa* (HKUCC 3713) clustered away from Savoryellaceae but within the subclass Savoryellomycetidae. However, *Monotosporella setosa* formed a basal lineage to the *Ascotaiwania hughesii* strains. They shared a sister clade relationship with Pleurotheciaceae (Figure 2).

### Molecular Clock Analysis

**FIGURE 3:**
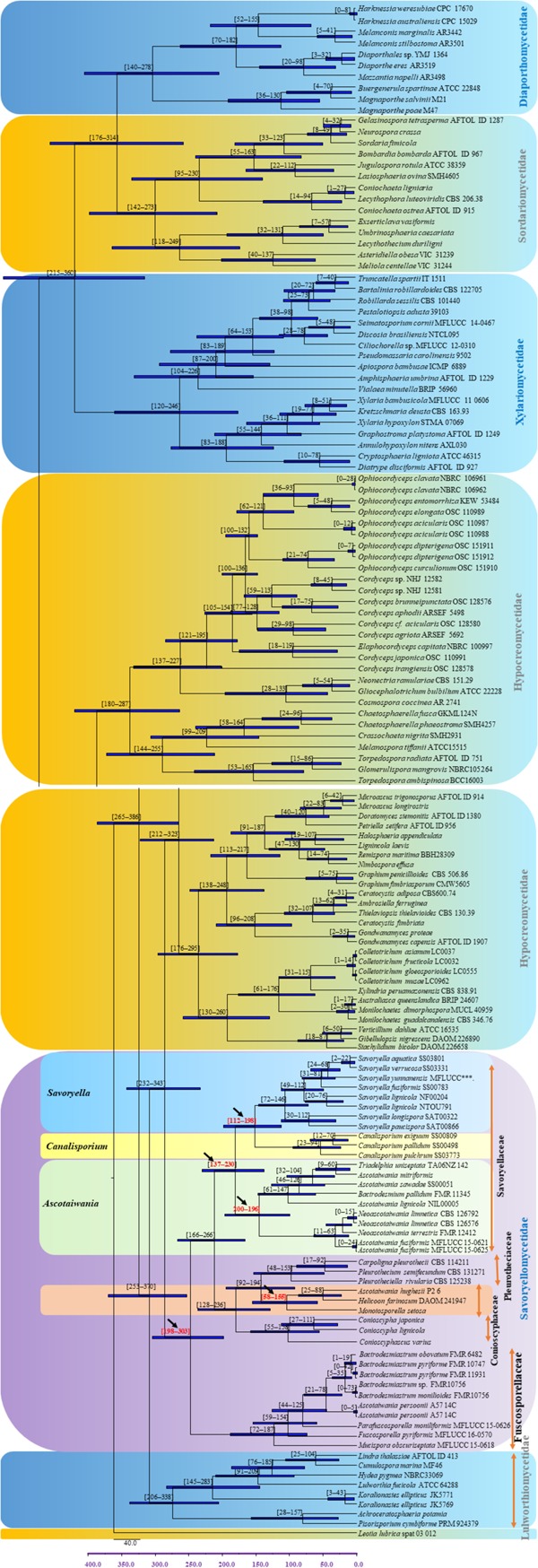
Maximum clade credibility (MCC) tree with divergence times estimates for Savoryellomycetidae using lognormal relaxed clock mode (uncorrelated) in BEAST, with representative families. Geological periods are indicated at the base of the tree.

**FIGURE 4:**
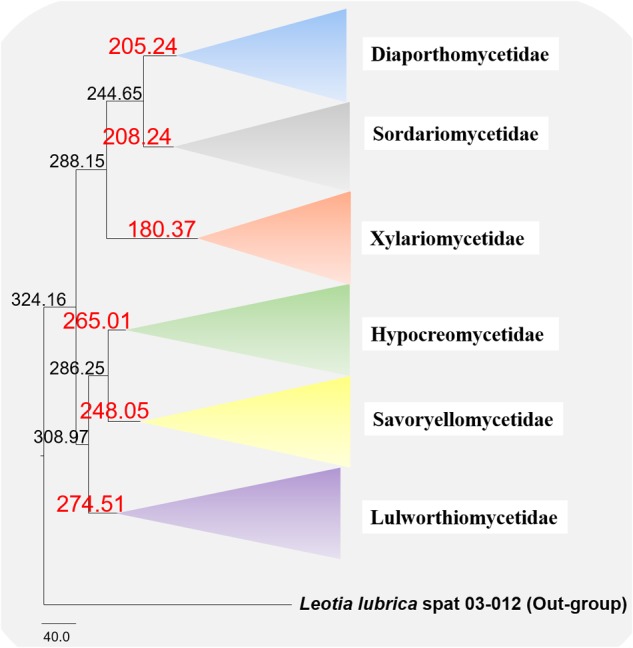
Maximum clade credibility (MCC) tree with divergence times estimates that indicates evolutionary ages of six subclasses estimated using lognormal relaxed clock mode (uncorrelated) in BEAST.

The evolutionary model based on sequence data linked to fossil data (Figure 3), reveals the divergence of Sordariomycetes at around 325 (265–386) Mya in the Paleozoic era. The major clades representing five sub-classes; Diaporthomycetidae, Hypocreomycetidae, Lulworthiomycetidae, Savoryellomycetidae, Sordariomycetidae, and Xylariomycetidae are well supported in both phylogenetic and the divergence time analyses with median crown ages of 206, 266, 274, 249, 208, 182 Mya, respectively (Figure 3, 4). Lulworthiomycetidae represents the basal clade with stem age of 310 (253–370) Mya that can be considered as the earliest diverged group comprising mostly marine originated taxa. The divergence of the Savoryellaceae crown group occurred 182 Mya, while the three genera *Savoryella, Ascotaiwania*, and *Canalisporium* evolved 56–108 Mya (Figure 3 and Table 3). Novel genus *Helicoascotaiwania* gen. nov. originated 53 (25–88) Mya while, other *Pleurotheciales* taxa evolved 98 (48–153) (Figure 3).

**Table 3:** Divergence time estimates of different taxa within Savoryellomycetidae from the present study.

Crown group	Crown age (Mya)
*Ascotaiwania*	146 (200–196)
*Canalisporium*	56 (23–94)
*Conioscyphaceae*	104 (55–158)
*Conioscyphales*	104 (55–158)
*Fuscosporellaceae*	126 (72–187)
*Fuscosporellales*	126 (72–187)
*Helicoascotaiwania*	53 (25–88)
*Pleurotheciaceae*	143 (92–194)
*Pleurotheciales*	143 (92–194)
*Savoryella*	108 (72–146)
Savoryellaceae	182 (137–230)
Savoryellales	182 (137–230)
Savoryellomycetidae	249 (198–303)

### Taxonomy

**Savoryellaceae** Jaklitsch & Réblová, in Jaklitsch (2015)
*Index Fungorum number*: IF 551026; *Facesoffungi number*: FoF 01283
*Description*: This family was treated in detail by Maharachchikumbura et al. (2016).
*Family type*: *Savoryella* E.B.G. Jones & R.A. Eaton (1969)

*Note*: Savoryellales taxa share a set of characters including immersed, semi-immersed to superficial, non-stromatic, heavily pigmented, coriaceous ascomata, mostly lying horizontally to the host, partly deliquescing, paraphyses, unitunicate asci comprises non-amyloid apical annulus, and fusiform to ellipsoidal, transversely septate ascospores with hyaline end cells and brown median cells (Jones and Eaton, 1969; Jones and Hyde, 1992; Tsui and Hyde, 2003; Jones et al., 2009; Boonyuen et al., 2011). Different types of asexual morphs (based on morphological observations only) have been experimentally linked to *Ascotaiwania* and *Neoascotaiwania* species such as, monotosporella-like in *A. sawada* (Sivichai et al., 1998) and *A. mitriformis* (Ranghoo and Hyde, 1998), monodictys-like in *A. lignicola* (Chang, 2001), trichocladium-like in *A. hsilio* (Chang, 2001), and bactrodesmium-like asexual morphs for *Neoascotaiwania* species (Hernández-Restrepo et al., 2017). These various asexual morphs have led to confusion in the classification of various sexual genera in the family. *Canalisporium* species comprise muriform conidia (sexual morph – Ascothailandia; Sri-indrasutdhi et al., 2010), but not all species have been sequenced. Therefore, Savoryellales asexual morphs are associated with dematiaceous hyphomycetes characterized by semi-macronematous conidiophores (conidiophores that are only slightly morphologically different from the vegetative hyphae) and monoblastic conidiogenous cells generating brown, thick-walled, dictyoseptate conidia that are transversely septate or cheiroid. The distant position of *Helicoon farinosum* (asexual morph of *Ascotaiwania hughesii*) (Fallah et al., 1999), from the rest of members of Savoryellales was confirmed by phylogenetic analysis (Boonyuen et al., 2011; Réblová et al., 2012).

***Savoryella*** E.B.G. Jones & R.A. Eaton (1969)
*Index Fungorum number*: IF 4870; *Facesoffungi number*: FoF 05391
*Description*: Please see Maharachchikumbura et al. (2016).
*Type species*: *Savoryella lignicola* E.B.G. Jones & R.A. Eaton (1969)

*Note*: *Savoryella* was introduced by Jones and Eaton (1969) and is typified by *S. lignicola*. *Savoryella* is a widespread genus of lignicolous ascomycetes in aquatic habitats. All species in the genus comprise ellipsoidal, three septate ascospores whose central cells are brown, and end cells are hyaline, with or without polar appendages (Jones et al., 2016). *Savoryella* was variously assigned to higher orders based on morphological observations: the order Sphaeriales family *incertae sedis* by Kohlmeyer and Kohlmeyer (1979); ascomycetes *incertae sedis* by Kohlmeyer (1986); Eriksson and Hawksworth (1986); Amphisphaeriaceae (Eriksson and Hawksworth, 1987) and Sordariales (Jones and Hyde, 1992). Barr (1990) established this genus within the order Halosphaeriales based on morphology (the catenophyses-like paraphyses) and ultrastructural observations, but these placements have all been rejected (Jones et al., 2016). *Savoryella elongata* and *S. longispora* referred to the order Hypocreales, subclass Hypocreomycetidae by Cai et al. (2006) and Vijaykrishna et al. (2006), based on a phylogenetic analysis of large subunit nuclear ribosomal DNA sequence data with low statistical support. Based on phenotypic characters, Jones et al. (2009) placed the *Savoryella* in Sordariales genera *incertae sedis*. Although the asexual morphs of *Savoryella* are still unknown (Boonyuen et al., 2011), dark brown, 3–5-septate conidia were observed from the host surface and in living cultures obtained from ascospore isolates of two *S. limnetica* strains (Chang et al., 1998). A later phylogenetic study by Réblová et al. (2016b), confirmed *S. limnetica* as an *Ascotaiwania* species. The genus currently comprises twelve species (Jones and Eaton, 1969; Hyde, 1994; Abdel-Wahab and Jones, 2000). *Savoryella limnetica* introduced by Chang et al. (1998) was latterly synonymized as *Neoascotaiwania limnetica* based on multi-gene analyses (Hernández-Restrepo et al., 2017). Key to *Savoryella* species is shown below.

#### Key to *Savoryella* Species

1. Asci 8-spored…………………………………………**2**
1. Asci 2-spored……………………………. ***S*. *paucispora***
2. Ascospores lack appendages…………………………. **3**
2. Ascospores with polar appendages…….. ***S*. *appendiculata***
3. Ascospores straight……………………………………**4**
3. Ascospores slightly curverd or curved, fusiform………………………………….. ***S. curvispora***
4. Ascospores with smooth walls……………………….. **5**
4. Ascospores with markedly verrucose walls…***S*. *verrucosa***
5. Ascospores often wider than 12 μm……………………**6**
5. Ascospores often narrower than 12 μm………………**7**
6. Ascospores ellipsoidal or irregular………***S*. *yunnanensis***
6. Ascospores ellipsoidal, brown or broadly ellipsoidal, dark brown………………………………………………**9**
7. Average width of ascospores <9 μm, freshwater species (ascospores 25 ± 35–6 ± 9 ± 6 μm) 27–32 × 12–17 μm………………………………………***S*. *fusiformis***
7. Average width of ascospores >8 ± 5 μm, intertidal or freshwater species……………………………………**8**
8. Mean length of ascospores <35 μm, intertidal or freshwater species (ascospores 24 ± 37 8 ± 14 μm)………***S*. *lignicola***
8. Mean length of ascospores >35 μm, intertidal species (ascospores 34 ± 47–7 ± 5 ± 12 μm)………***S*. *longispora***
9. Ascospores ellipsoidal, brown…………………………**10**
9. Ascospores broadly ellipsoidal, dark brown.. ***S. grandispora***
10. Found in marine habitat, ascomata (160–305 × 184–225 μm), asci (170–212 × 15–25), and ascospores (32–45 × 15–18 μm)…………………………………***S. melanospora***
10. Found in freshwater habitat ascomata (195–260 × 91–130 μm), asci (106–140 × 26–34), and ascospores (29–38 × 13.5–17 μm)…………………………***S*. *aquatica***

Sequence data are available only for *S. aquatica, S. fusiformis, S. lignicola, S. longispora, S. verrucosa*, and *S. paucispora*. Phylogenetic analyses showed *Savoryella* members formed a well-defined (ML 100%, 1.00 PP) clade within order Savoryellales (Boonyuen et al., 2011; Jones et al., 2016; Hernández-Restrepo et al., 2017).

***Savoryella lignicola*** E.B.G. Jones & R.A. Eaton (1969), Figure 5
*Index Fungorum:* IF 338840; *Facesoffungi number:* FoF 05392

**FIGURE 5:**
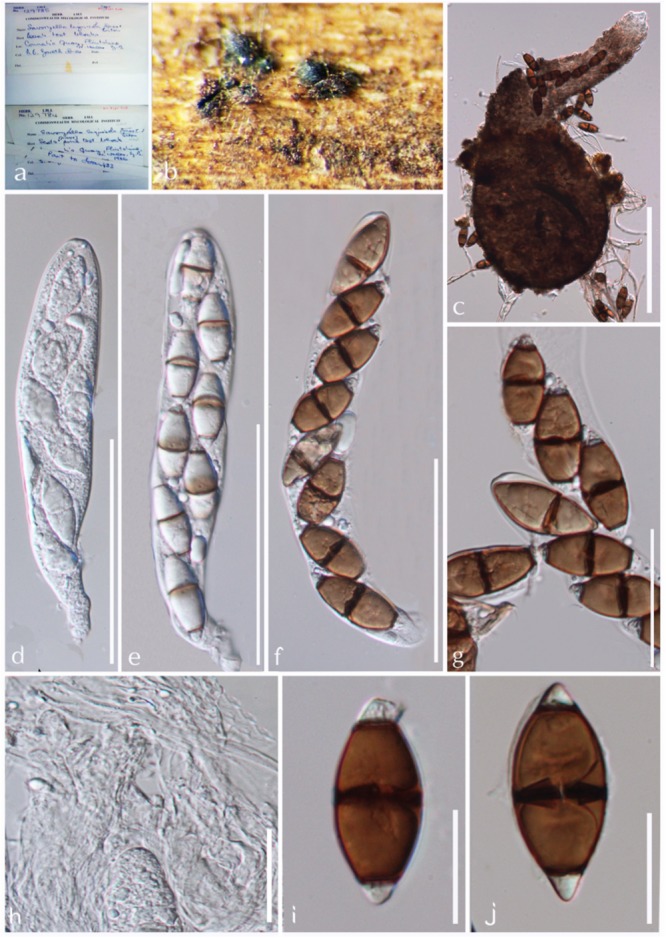
*Savoryella lignicola* (IMI 129784, IMI 129785, holotype). **(a)** Herbarium specimen. **(b,c)** Ascomata on host. **(d,e)** Ascomata. **(f)** Paraphyses. **(g–i)** Asci. **(j–n)** Ascospores. Scale bars: **(c)** 200 μm, **(d,e)** 100 μm, **(g–i)** 50 μm, **(j–n)** 20 μm.

*Saprobic* on submerged wood in water cooling towers, rivers, streams, and marine environments. **Sexual morph**: *Ascomata* 180–320 × 120–150 μm perithecoid, subglobose to globose or ellipsoidal, superficial, semi-immersed or immersed, ostiollate, papillate, membranous, light to dark brown, with a long-neck, up to 80–165 μm, brown, periphysate. *Peridium* brown, composed of several layers of thick-walled angular cells forming *textura angularis*. *Paraphyses* present, but sparse. *Asci* 128–180 × 16–24 μm, 2–8-spored, cylindrical or clavate, short-pedicellate, unitunicate, persistent, with an apical truncate non-amyloid apical ring with a pore. *Ascospores* 20–34 × 8–12 μm, 1–2 seriate, ellipsoidal, 3-septate, not distinctly constricted at the septa, central cells brown, apical cells smaller and hyaline. **Asexual morph**: Undetermined (modified description of Maharachchikumbura et al., 2016).

*Material examined:* United Kingdom, Flintshire, Connah’s Quay, on Scots pine test-blocks placed for 168 days amongst the packing timber of a water-cooling tower at Connah’s Quay, 16 June 1966 and 1 December 1966, IMI 129784, IMI 129785 type.

*Note*: *Savoryella lignicola* was initially isolated from a water-cooling tower in Great Britain and has now been reported as a cosmopolitan species (Jones and Eaton, 1969; Ho et al., 1997; Jones et al., 2016). This is the sole *Savoryella* taxon detailed from both marine and freshwater environments (Ho et al., 1997; Luo et al., 2004). Though the marine and freshwater isolates of *S. lignicola* are morphologically alike, it is doubtful whether they are same species (Ho et al., 1997). Molecular data are available only for two *S. lignicola* strains described from mangrove wood from Malaysia and submerged *Nypa fruticans* fronds from Thailand, with no molecular data for the freshwater strain of *S. lignicola* (Ho et al., 1997; Boonyuen et al., 2011). Therefore, molecular data should be obtained from collections from freshwater habitats to establish whether they are the same species or not. *Savoryella lignicola* morphologically resembles *S. fusiformis* and *S. longispora* (Boonyuen et al., 2011). However, these taxa can easily be distinguished by measurements of length/width ratio of ascospores and molecular data (Ho et al., 1997; Boonyuen et al., 2011).

***Savoryella aquatica*** K.D. Hyde (1993)
*Index Fungorum*: IF 361052; *Facesoffungi number*: FoF 05393
*Description*: Please see Jones and Hyde (1992).
*Material examined*: Australia, Atherton Tablelands, Koah, Clohesy River, on submerged wood, October 1990, K.D. Hyde, BRIP 19327, holotype.

*Note*: *Savoryella aquatica* differs from *S. lignicola* and *S. longispora*, by having wider ascospores (13.5–17 μm vs. 7.5–12 μm). It differs from *S. appendiculata*, which has appendaged ascospores, *S. paucispora*, with two-spored asci, and *S. verrucosa* that have distinctly verrucose ascospore walls (Jones and Hyde, 1992). However, no ascomata of *S. aquatica* were present in the type material of the species. Therefore, epitypification is needed for future studies.

***Savoryella grandispora*** K.D. Hyde (1994), Figure 6
*Index Fungorum*: IF 362061; *Facesoffungi number*: FoF 05394

**FIGURE 6:**
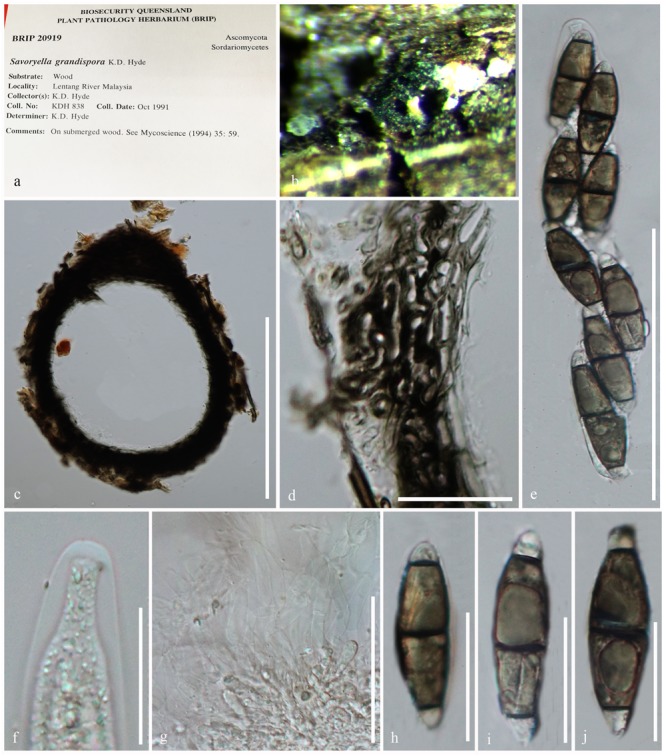
*Savoryella grandispora* (BRIP 20918, holotype). **(a)** Herbarium specimen. **(b)** Ascomata on host. **(c)** Section through an ascoma. **(d)** Peridium. **(e)** Ascus. **(f)** Apical ring. **(g)** Paraphyses. **(h–j)** Ascospores. Scale bars: **(c)** 200 μm, **(e)** 50 μm, **(d,g)** 20 μm, **(f–j)** 10 μm.

*Ascomata* 200–250 × 90–135 μm, immersed or superficial, coriaceous, pyriform, brown to dark brown, papillate, solitary or gregarious. *Necks* cylindrical, brown, pale and slightly tapering toward the apex, periphysate. *Peridium* 20–40 μm wide, thin, brown, of *textura epidermoidea*. *Paraphyses* sparse, septate. *Asci* 80–100 × 12–16 μm, 8-spored, cylindrical, pedicellate, unitunicate, persistent, with a J-apical ring. *Ascospores* 22–28 × 5–8 μm, uniseriate, fusiform, curved, 3-septate, slightly constricted at the septa, smooth, thin-walled; central cells brown, hyaline to pale brown (description generated from herbarium material observation and observations of Ho et al., 1997).

*Material examined*: Malaysia, State Negara, Lipur Lentang Nature Reserve, on submerged wood, October 1991, K.D. Hyde, BRIP 20918, holotype.

*Note*: *Savoryella grandispora* is a distinctive species in the genus with the largest ascospores (46–58 × 14–16 μm) among *Savoryella* species (Hyde, 1993, 1994). *Savoryella grandispora* has similar morphological features to *S. aquatica* (Hyde, 1993). *Savoryella grandispora* is distinguished in having longer ascospores which are light-brown as compared to the dark brown ascospores of *S. aquatica* (Hyde, 1994). However, the sequence data are unavailable for *S. grandispora* to compare their phylogenetic affinities.

***Savoryella longispora*** E.B.G. Jones & K.D. Hyde (1992), Figure 7
*Index Fungorum*: IF 358210; *Facesoffungi number*: FoF 05395

**FIGURE 7:**
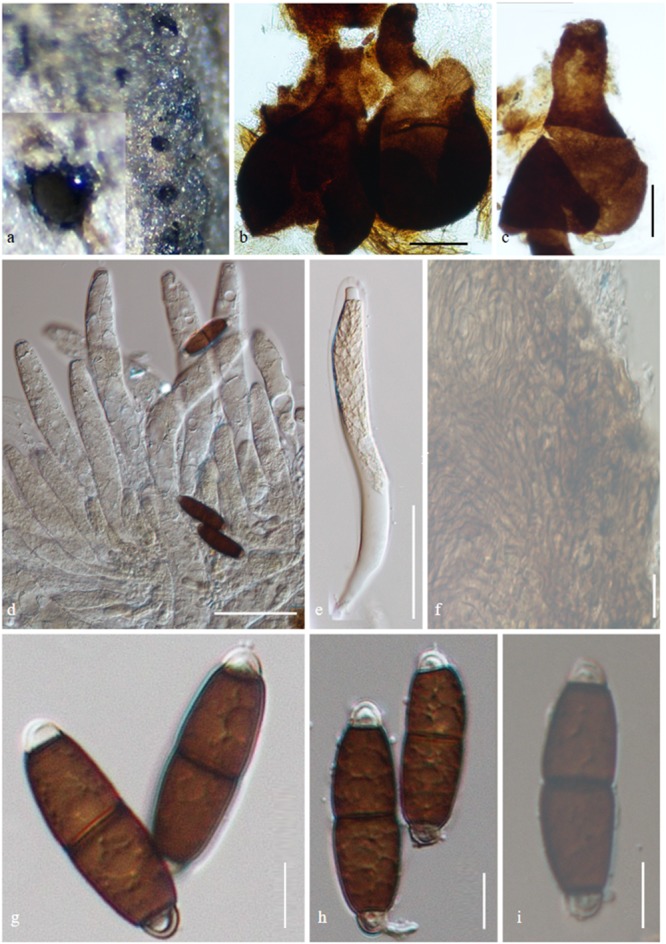
*Savoryella longispora* (AMH-9997, isotype). **(a)** Ascomata semi-immersed on decaying wood of *Avicennia marina.* **(b,c)** Squash mounts of ascomata. **(d,e)** Immature asci. **(f)** Peridium. **(g–i)**. Ascospores. Scale bars: **(b,c)** 100 μm, **(d,e)** 50 μm, **(f–i)** 10 μm.

*Saprobic* on decaying wood of *Avicennia marina*. *Ascomata* 175–340 × 205–250 μm ($\bar{X}$ = 341 × 231 μm, *n* = 5), globose to subglobose or ellipsoidal, immersed, semi-immersed or superficial, ostiolate, papillate, membranous, and light to dark brown. *Necks* 115–160 × 55–60 μm ($\bar{X}$ = 137 × 64 μm, *n* = 5), brown, with periphyses. *Peridium* 10–25 μm ($\bar{X}$ = 18, *n* = 5), brown, one-layered, comprises several layers of thick-walled angular cells forming *textura angularis. Paraphyses* present, sparse. *Asci* 150–200 × 15–25 μm, ($\bar{X}$ = 245 × 186 μm, *n* = 15), 8-spored, cylindrical or clavate, short-stalked, unitunicate, persistent, with truncate, J-apical ring containing a pore. *Ascospores* 34–45 × 7.5–12 μm ($\bar{X}$ = 39 × 10 μm, *n* = 5), uni- or biseriate, ellipsoidal, 3-septate, slightly constricted at the septa, central cells brown, apical cells smaller and hyaline (description generated from observation of herbarium material and the description of Jones and Hyde, 1992).

*Material examined*: INDIA: Tamil Nadu, Muthupet mangroves (10.4°N 79.5°E), on decaying wood of *Avicennia marina* (Acanthaceae), 30 March 2016, B. Devadatha (AMH-9997).

*Note*: *Savoryella longispora* was first reported from intertidal mangrove wood from Malaysia (Jones and Hyde, 1992). Both *Savoryella longispora* and *S. lignicola* share similar morphological characteristics but *S. longispora* is distinguished in having longer (34–45 μm vs. 24–36 μm) and narrower ascospores (7.5–12 μm vs. 8–12 μm) when compared to *S. lignicola* (Abdel-Wahab and Jones, 2000).

**FIGURE 8:**
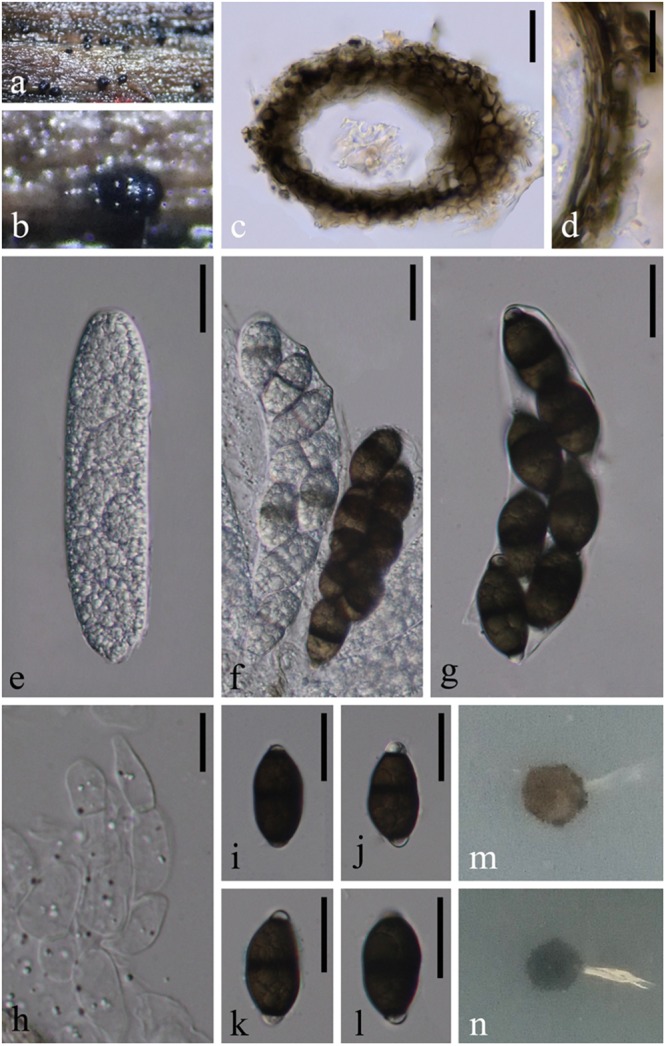
*Savoryella yunnanensis* (MFLU 18-1203, holotype) **(a,b)** Appearance of black ascomata superficial on host. **(c)** Vertical section of ascoma. **(d)** Structure of peridium. **(e–g)** unitunicate asci. **(h)** Paraphyses. **(i–l)** Ascospores. **(m)** Colony on PDA (from front). **(n)** Colony on PDA (from below). Scale bars: **(c,e–g,i–l)** 20 μm, **(d,h)** 10 μm.

***Savoryella yunnanensis*** W. Dong, Dayarathne & K.D. Hyde **sp. nov.**, Figure 8
*Index Fungorum*: IF555621; *Facesoffungi number*: FoF 05484
*Etymology*: Species epithet derived according to the geographical location, Yunnan province where the taxon found.

*Saprobic* on decaying wood submerged in freshwater. **Sexual morph**: *Ascomata* 70–90 μm high, 110–130 μm diameter, black, scattered or solitary, superficial, ellipsoidal, unilocular, thin-walled, laterally ostiolate, lying horizontal to the host surface with a short, hyaline neck. *Peridium* 15–20 μm thick, comprising several layers of dark brown to brown, thick-walled cells of *textura angularis*. *Paraphyses* ca. 8 μm diameter, numerous, swollen cylindrical, unbranched, hyaline, septate, constricted at the septa. *Asci* 100–135 × 25–30 μm ($\bar{X}$ = 115 × 27 μm, *n* = 10), 8-spored, unitunicate, clavate to narrow ellipsoidal, with short or without pedicellate. *Ascospores* 27–32 × 12–17 μm ($\bar{X}$ = 30 × 15 μm, *n* = 15), biseriate, straight or slightly curved, hyaline when young, central cells dark brown when old, end cells hyaline, 1-septate, slightly constricted at the septa, ellipsoidal or irregular, thin-walled, without sheaths or appendages. **Asexual morph**: Undetermined.

*Culture characteristics*: On PDA, colony circular, reaching 5 mm in 45 days at 25°C, gray to brown from above, gray from below, surface rough, dry, edge entire.

*Material examined*: China, Yunnan Province, Dehong, on submerged wood in a stream, 25 November 2017, W. Dong, H40C (MFLU 18-1203, holotype), ex-type living culture, MFLUCC 18-1395; on submerged wood in a small river, 25 November 2017, W. Dong H40C (HKAS 101726, isotype), living culture KUMCC 18-0076.

*Note*: *Savoryella yunnanensis* is morphologically most similar to *Savoryella lignicola*. However, it differs by possessing shorter and wider asci (128–180 × 16–24 μm vs. 100–135 × 25–30 μm) and wider ascospores (24.5–33.5 × 8.5–12.5 μm vs. 27–32 × 12–17 μm) and the shape of ascospores (ellipsoidal vs. ellipsoidal or irregular) (Jones and Eaton, 1969). *Savoryella yunnanensis* formed a well-supported (65% ML) separate lineage basal to a clade comprising *Savoryella aquatica* and *Savoryella verrucosa*. Base pair difference of *S. yunnanensis* and *S. verrucosa* for LSU only 4 bp out of 940 bp (<1%), SSU 30 bp out of 1,073 bp (2.79%), RPB2 116 bp out of 964 (12.03%) which are in the required range to consider them as two distinct species.

#### Other Genera Accepted in Savoryellaceae

***Ascotaiwania*** Sivan. & H.S. Chang (1992)
*Index Fungorum*: IF 25163; *Facesoffungi number*: FoF 05693
= ***Neoascotaiwania*** Hern.-Restr., R.F. Castañeda & Guarro (2017)

*Saprobic* on decaying plant parts including wood, petioles, twigs, prop roots. **Sexual morph**: *Ascomata* singled to grouped, partly to completely immersed in the host substrate, oblique or horizontal, dark brown to black globose, with a lateral short to long, erect, periphysate beak. *Peridium* composed of pseudoparenchymatous cells. *Paraphyses* filiform, hyaline, simple to rarely branched, deliquescing early. *Asci* cylindrical, 8-spored, pedicellate, functionally unitunicate, with a distinct non-amyloid apical annulus. *Ascospores* uniseriate to overlapping biseriate in the ascus, fusoid, transversely 5- or 3- or 7-septate, not constricted, straight to somewhat curved, smooth, with larger brown central cells and smaller hyaline to subhyaline end cells (modified description of Sivanesan and Chang, 1992). **Asexual morph**: brachysporiella-like, bactrodesmium-like, monodictys-like or monotosporella-like or trichocladium-like. *Conidiophores* micronematous to semi-macronematous or mononematous, flexuous, smooth, thin-walled, hyaline or thick walled and light brown to brown, occasionally indistinct. *Conidiogenous cells* erect or prostrate, holoblastic or monoblastic, integrated or determinate, mostly intercalary or terminal directly differentiate and developed at random point regions of hyphae, sometimes septate, smooth and thick-walled, cylindrical, flask-shaped, pale to dark brown some are arising from brown 2- or 3-septate rhizoids. *Conidia* solitary, frequently aggregate, globose, subglobose to obpyriform or ellipsoidal or subspherical, some aggregates, 1–4 septate, slightly constricted at the septa or constricted at the septa, sometimes a prominent scar on the conidium at the point of session from the conidiogenous cell, dark reddish brown to almost dark, smooth, the basal cells are mid-brown while the upper cell is blackish brown to black (modified description of Rao and de Hoog, 1986).

*Type species*: *Ascotaiwania lignicola* Sivan. & H.S. Chang (1992)

*Note*: *Ascotaiwania* was introduced by Sivanesan and Chang (1992) for an ascomycete with dark-brown to black ascomata having short or long lateral necks, asci with a comparatively massive J-apical ring and 5- or 3- or 7-septate pigmented ascospores with hyaline polar cells with *A. lignicola* as the type species. The family placement of *Ascotaiwania* has been widely debated (Ranghoo et al., 1999; Hernández-Restrepo et al., 2017). *Ascotaiwania* currently comprises 12 species. Initially, *Ascotaiwania* comprised taxa with only 7-septate ascospore (Sivanesan and Chang, 1992). Later, taxa with 3- or 5-septate ascospores were accepted in the genus, i.e., *A. hsilio, A. hughesii, A. palmicola, A. pallida, A. pennisetorum, A. persoonii*, and *A. sawada* (Chang et al., 1998) and subsequently, *A. mauritiana, A. mitriformis*, and *A. wulai* (Ranghoo and Hyde, 1998; Fallah et al., 1999; Hyde and Goh, 1999; Hernández-Restrepo et al., 2017). However, *A. persoonii* was synonymized under *Pseudoascotaiwania persoonii* within the order Fuscosporellales by Yang et al. (2016).

**FIGURE 9:**
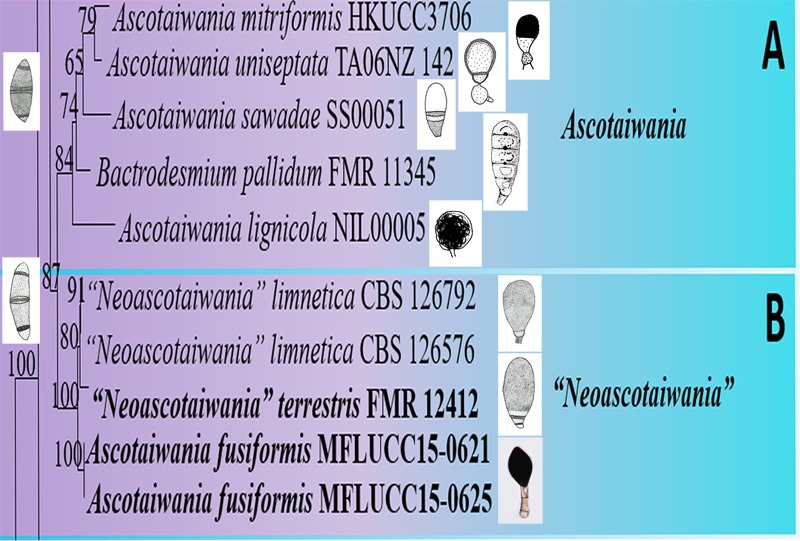
Conidial and ascospore character comparison of **(A)** *Ascotaiwania* and **(B)** “*Neoascotaiwania*” species.

**FIGURE 10:**
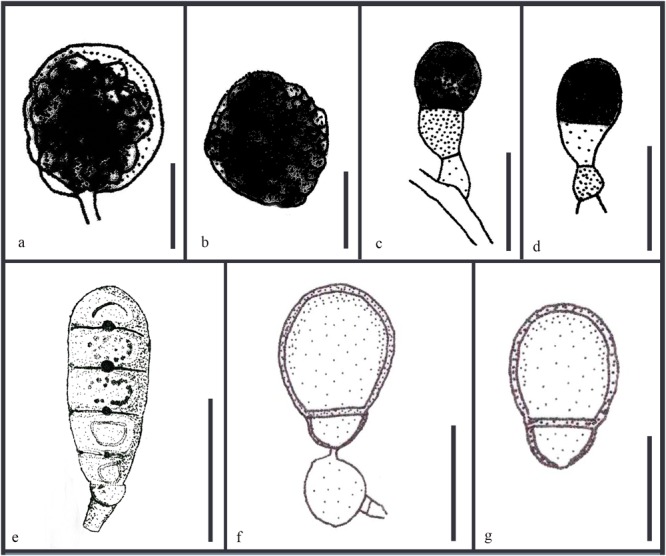
Revised drawings of different asexual morphs of *Ascotaiwania*. Conidiogenous cells and conidia of **(a,b)** *A. lignicola* (Chang, 2001) **(c,d)** *A. mitriformis* (Ranghoo and Hyde, 1998) **(e)** *B. pallidum* (Tsui and Hyde, 2003) **(f,g)** *A. uniseptata* (Kirk, 1983). Scale bars: **(a,b,e)** 20 μm, **(c,d,f,g)** 10 μm.

Asexual morphs of *Ascotaiwania* species have different morphologies (Hernández-Restrepo et al., 2017), such as monodictys-like comprising multicellular dark brown conidia in *A. lignicola* (Chang, 2001) (Figure 10); trichocladium-like, 1-septate conidia, which have dark brown apical cells and smaller, hyaline basal cells in *A. hsilio* (Chang, 2001); monotosporella-like in taxa in *A. mitriformis* (Ranghoo and Hyde, 1998) and *A. sawada* (Sivichai et al., 1998); and *Helicoon farinosum* in *A. hughesii* (Fallah et al., 1999). Jones et al. (2015), Réblová et al. (2016b), and Hernández-Restrepo et al. (2017) established that *Ascotaiwania* is polyphyletic and their asexual morph is phylogenetically more informative than the sexual morph. Recent studies by Yang et al. (2016) revealed *Ascotaiwania fusiformis* (asexual morph), clustered as a sister species to *A. limnetica* (=*Neoascotaiwania limnetica*). In our phylogenetic analyses, it grouped more closely to *Neoascotaiwania terrestris*, an asexual morph introduced by Hernández-Restrepo et al. (2017). Hernández-Restrepo et al. (2017) differentiated *Neoascotaiwania* from *Ascotaiwania* in having 3-septate ascospores, asci with a thinner, non-amyloid apical ring, and a bactrodesmium-like asexual morph. Phylogenetic analysis with a larger number of taxa (Figure 2) shows that both *Neoascotaiwania* and *Ascotaiwania* cluster in a single clade with high statistical support (100% ML, 1.00 PP). Two subclades (A and B in Figure 2) can be recognized within this clade. However, both clades comprise species with overlapping morphologies, i.e., *Neoascotaiwania limnetica* (subclade B) which is the type of *Neoascotaiwania* has three septate ascospores, while *A. sawada* also has three septate ascospores (subclade A) (Figure 9). Further, species with bactrodesmium-like asexual morphs, such as *Ascotaiwania uniseptata, Bactrodesmium pallidum, Neoascotaiwania terrestris* are distributed in both subclades (Figure 9). Therefore, we believe that species morphologies of both of those subclades are insufficient to distinguish them as two distinct genera, hence, we synonymize *Neoascotaiwania* under *Ascotaiwania*.

Asexual morph *Bactrodesmium pallidum* nested in subclade B (Figure 2) with, *A. mitriformis, A. sawadae, A. lignicola* and *Triadelphia uniseptata*. *Ascotaiwania mitriformis* and *A. sawadae* showed monotosporella-like asexual morphs (Ranghoo and Hyde, 1998) while, *A. lignicola* (Chang, 2001) has trichocladium-like asexual morphs (Figure 10). *Triadelphia uniseptata* resembles *B. pallidum* in producing holoblastic, brown, septate conidia but *B. pallidum* differs by having sporodochial conidiomata and slightly differentiated, hyaline conidiophores (Holubová-Jechová, 1972; Tsui and Hyde, 2003; Réblová et al., 2016a). However, both *B. pallidum* and *Triadelphia uniseptata* clustered with *Ascotaiwania* species. According to both morpho-molecular data, this was supported by previous studies of Boonyuen et al. (2011) and Réblová et al. (2016b). Among *Helicoon* species, only *Helicoon farinosum* has been referred to the Savoryellaceae and it has been experimentally linked as the asexual morph of *Ascotaiwania hughesii* by rDNA data (Fallah et al., 1999). The *Monotosporella* state of *Ascotaiwania sawada* and *A. mitriformis* were regarded as different species from *M. setosa* and were regarded as two different taxa (Ranghoo and Hyde, 1998; Sivichai et al., 1998). In the analysis of the large subunit nuclear ribosomal DNA sequences by Ranghoo et al. (1999) they were separated as individual species, who established the connection between *Ascotaiwania* and *Monotosporella*. While later analyses with a higher number of taxon sampling (Boonyuen et al., 2011; Yang et al., 2016; Hernández-Restrepo et al., 2017) showed that *M. setosa* clustered away from the *Ascotaiwania* clade and formed a basal lineage to *A. hugesii* (P2-6, DAOM 241947) within Pleurotheciales. This is supported in our study (Figure 2, 3). We exclude *A. hugesii* and *Monotosporella setosa* from *Ascotaiwania*. Therefore, the genus *Ascotaiwania* is no longer polyphyletic.

***Ascotaiwania fusiformis*** Jing Yang, Bhat & K.D. Hyde (2016), Figure 11
*Index Fungorum*: IF 552294; *Facesoffungi number*: FoF 02429
*Description*: Please see Yang et al. (2016).

**FIGURE 11:**
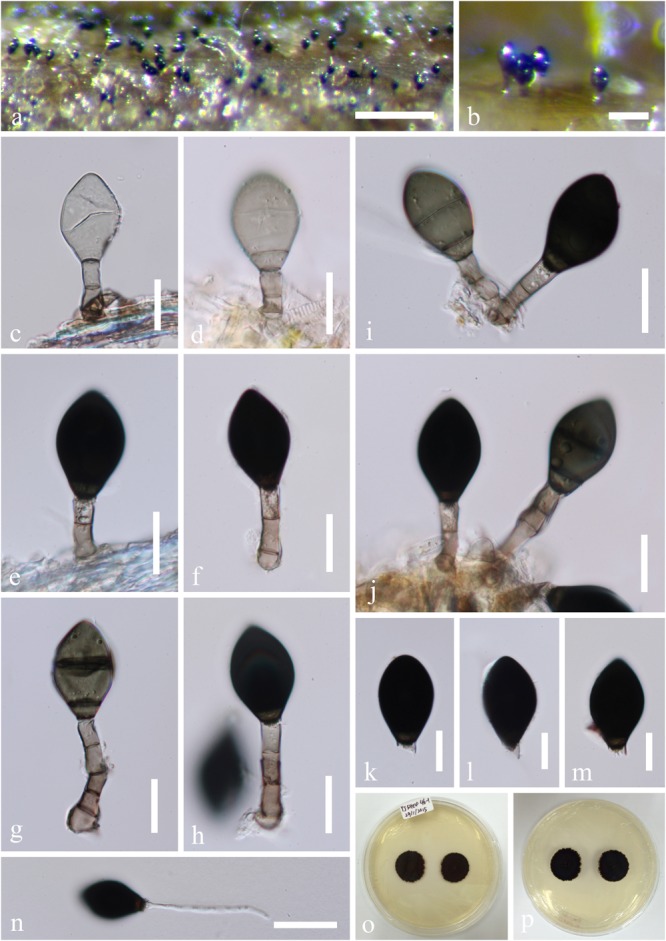
*Ascotaiwania fusiformis* (MFLU15-1156, holotype). **(a)** Colonies on the substrate. **(b–g)** Conidiophores, developing and mature conidia. **(h–k)**. Conidiophores and conidiogenous cell. (**l–q)** Conidia. **(r)** Germinated conidium on PDA. **(s,t)** Culture, from above **(s)**, from below **(t)**. Scale bars: **(a)** 50 μm, **(b)** 15 μm, **(c–k)** = 20 μm, **(l–q)** 15 μm, **(r)** 40 μm.

*Material examined*: Thailand, Prachuap Khiri Khan Province, on decaying submerged wood in a stream, 25 December 2014, Jaap van Strien, Site4- 27-1, MFLU 15-1156, holotype, ex-type living culture, MFLUCC 15-0621; *ibid*., HKAS 95048, isotype.

*Note*: In our phylogenetic analyses, *Ascotaiwania fusiformis* grouped in a clade comprising *A. limnetica* and *A. terrestris*. *Ascotaiwania fusiformis* morphologically resembles *Monotosporella* species and the brachysporiella-like asexual morphs of *Ascotaiwania* (Sadowski et al., 2012; Réblová et al., 2016b). *Ascotaiwania fusiformis* is easily distinguishable from *Monotosporella rhizoidea* in the absence of lobe-like swollen conidiogenous cells (Rao and de Hoog, 1986), while it can be separated from *Monotosporella clavata* which has clavate, 4–6-septate conidia, by it’s fusiform and less septate conidia (Yanna and Hyde, 2002). *Ascotaiwania fusiformis* differs from *Monotosporella doerfeltii* by larger conidia with dark basal cells (Sadowski et al., 2012). *Brachysporiella*-like asexual morphs of *A. limnetica* have (3–) 5–6 septate conidia, while *A. fusiformis* usually has 2-septate conidia (Réblová et al., 2016b). *Ascotaiwania terrestris* morphologically differs from *A. fusiformis* in having (2–)3–4(–5)- septate, ellipsoidal to obovoid conidia (Hernández-Restrepo et al., 2017).

***Ascotaiwania limnetica*** (Hern.-Restr., R.F. Castañeda & Gené) Dayarathne, Hyde KD **comb. nov**.
*Index Fungorum*: IF555623
*Basionym*: *Savoryella limnetica* H.S. Chang & S.Y. Hsieh, in Chang, Hsieh, Jones (1998)
*Synonym*: *Ascotaiwania limnetica* (H.S. Chang & S.Y. Hsieh) Réblová & J. Fourn., in Réblová, Seifert, Fournier & Štìpánek (2015) [2016]

*Descriptions and illustrations*: Hernández-Restrepo et al. (2017)

***Ascotaiwania terrestris*** (Hern.-Restr., R.F. Castañeda & Guarro) Dayarathne, Hyde KD **comb. nov.**
*Index Fungorum*: IF555622
*Basionym*: *Neoascotaiwania terrestris* Hern.-Restr., R.F. Castañeda & Guarro, in Hernández-Restrepo et al. (2017)
*Descriptions and illustrations*: Chang et al. (1998) and Réblová et al. (2016b).

***Ascotaiwania uniseptata*** (Berk. & Broome) P.M. Kirk (1983), Figure 10
*Index Fungorum*: IF 109271
*Basionym*: *Sporidesmium uniseptatum* Berk. & Broome (1859)
*Description and illustrations*: Please see Kirk (1983)

*Note*: In our phylogram, *Ascotaiwania uniseptata* (=*Triadelphia uniseptata*), a dematiaceous hyphomycete, clustered in the *Ascotaiwania* clade with affinities to *A. mitriformis.* This placement was supported by the previous studies of Réblová et al. (2016b), Yang et al. (2016), and Hernández-Restrepo et al. (2017). The genus *Triadelphia* typified by *T. heterospora*, was introduced to include fungi from freshwater and brackish water habitats (Shearer and Crane, 1971). *Triadelphia heterospora* is characterized by sub globose to subglobose, subhyaline to dematiaceous conidiogenous cells, schizolytic conidial secession while conidia generated blastically from a single locus, brown, or versicolorous conidia often with one or two end cells which are lighter than the middle ones, septate, often with brighter bands masking some septa (Shearer and Crane, 1971). *Triadelphia heterospora* was described with dimorphic conidia (Constantinescu and Samson, 1982). There are 22 species records in Index Fungorum while one species was synonymized under *Pithomyces*^3^. Phylogenetic position of its type species is unknown. Only a few species of *Triadelphia*; *T. heterospora, T. pulvinata, T. moubasheri* have molecular data in GenBank and they have affinities to species in the Microascales (Edathodu et al., 2013; Wijayawardene et al., 2017, 2018). *Triadelphia* species resemble *Bactrodesmiastrum* in having cylindrical to lageniform, aggregated; conidiogenous cells (Réblová et al., 2016b). The morphology of *Ascotaiwania uniseptata* is illustrated in Figure 10. Réblová et al. (2016b) referred *Ascotaiwania uniseptata* to the Savoryellales and this is supported by Yang et al. (2016), who demonstrated that *Triadelphia* is polyphyletic. The continuation of this generic name, and inclusion of its species within the genus, needs additional sampling. However, *T. heterospora*, the generic type has no sequence data existing in GenBank. By considering the morphological similarities and phylogenetic togetherness, Boonyuen et al. (unpublished) proposed the inclusion of this species under *Ascotaiwania* and introduced the new family Triadelphialaceae to accommodate other *Triadelphia* species.

***Bactrodesmium pallidum*** M.B. Ellis (1959), Figure 10
*Index Fungorum*: IF 293575
*Basionym*: *Bactrodesmium pallidum* M.B. Ellis (1959)
*Description and illustrations*: Please see Tsui and Hyde (2003)

*Note*: *Bactrodesmium* is typified by *B. abruptum* isolated from dead wood in the United Kingdom (Berkeley and Broome, 1865). The taxonomy of the genus is doubtful and presently known to be polyphyletic (Hernández-Restrepo et al., 2013). It has been placed in several orders and families: Helotiales (Koukol and Koláøová, 2010), Massarineae (Tanaka et al., 2015) and currently placed in Pleosporales genera *incertae sedis* (Wijayawardene et al., 2018). However, there is no designated type material or living culture for the type species. Furthermore, there is a limited number of cultures available (Hernández-Restrepo et al., 2013). Tsui and Hyde (2003) described the morphological characters of *B. pallidum* isolated from wood submerged in a river in Japan (Figure 10). There are 61 species recorded under *Bactrodesmium* while five species have synonyms under *Melanommataceae* and *Magnaporthaceae*^4^. Therefore, the bactrodesmium-like taxa are polyphyletic. Phylogenetic analyses by Hernández-Restrepo et al. (2013) showed that *B. pallidum* grouped with *Ascotaiwania* and this was supported in our study. Hence, depending on present morphological and molecular data, we suggest the accommodation of *B. pallidum* within *Ascotaiwania*.

#### Key to Species of *Ascotaiwania*

1. Asexual morphs, bactrodesmium-like or brachysporiella-like, monodictys-like, monotosporella-like or trichocladium-like……………………………………. **2**
1. Sexual morphs only……………………………………**6**
2. Asexual morphs, bactrodesmium-like, brachysporiella-like or triadelphia-like……………………………………. **3**
2. Asexual morphs, monodictys-like, monotosporella-like or trichocladium-like……………………………………. **5**
3. Conidia fusiform, obovoid to broadly obovoid, 1–2-septate at all the stages……………………………………….. **4**
  3. Conidia ellipsoidal, obovoid, mostly 3–4-septate……………………………………***A. terrestris***
4. Conidia 1-septate near the base, constricted at the septum, upper cell dark and thick-walled, lower cell thin-walled……………………………………***A. uniseptata***
4. Conidia 2-septate, seen as uniseptate when mature, not constricted at the septum, subhyaline when young, becoming olive to dark brown, paler at the basal cell………………………………………***A. fusiformis***
5. Asexual morphs, monodictys-like……………***A. lignicola***
5. Asexual morphs, trichocladium-like……………***A. hsilio***
5. Asexual morphs, monotosporella-like…………………………………………***A. sawada***
6. Ascospores with polar appendages…………***A. palmicola***
6. Ascospores without polar appendages…………………**4**
7. Ascospores 3-septate………………………………….. **5**
7. Ascospores 5–7-septate……………………………….. **7**
8. Ascospores slightly constricted at the septa…………….. **6**
8. Ascospores not constricted at the septa…***A. pennisetorum***
9. Peridium comprising *textura intricata*, hyaline at the inside and brown outwardly, fusing at the outside with the host cells, composed of brown thick walled *textura angularis* ………………………………………………***A. pallida***
9. Peridium with opaque walls and sparse pores, the inner layer comprising thin-walled, hyaline, flattened cells, outward grading into small protruding cells, outer layer consisting of dark brown, polyhedral, flattened cells of *textura prismatica*…………………………***A. limnetica***
10. Ascospores shorter than 40 μm (19–30 μm). ***A. mauritiana***
10. Ascospores 55 μm or longer (53 ± 62 μm)………***A. wulai***

***Canalisporium*** Nawawi & Kuthub. (1989)
= ***Ascothailandia*** Sri-indr., Boonyuen, Sivichai & E.B.G. Jones (2010)
*Index Fungorum*: IF 11041; *Facesoffungi number*: FoF 05485
*Description:* Please refer Goh et al. (1998) and Sri-indrasutdhi et al. (2010).
*Type species*: *Canalisporium caribense* (Hol.-Jech. & Mercado) Nawawi & Kuthub. (1989)
*= Berkleasmium caribense* Hol.-Jech. & Mercado (1984)

*Note*: *Canalisporium* species are characterized by having scattered, punctiform, pulvinate, granular, black, glistening sporodochia, which contain acrogenous, holoblastic conidia that developed in a hyaline gelatinous sheath (Goh et al., 1998). Conidia of *Canalisporium* species are muriform, however, they differ from those of *Berkleasmium* species in being flattened dorsoventrally, comprising a single layer of regularly arranged cells, which are supported by a small, thin-walled, cuneiform, pale basal cell (Sri-indrasutdhi et al., 2010). At present, 15 species are recognized in *Canalisporium* (Zhao et al., 2012; Hyde et al., unpublished). The monotypic genus *Ascothailandia* was established by Sri-indrasutdhi et al. (2010) for the sexual morph of *C. grenadoideum* in a phylogenetic tree based on six species of *Canalisporium*, with the type species, and confirmed that *Ascothailandia* and *Canalisporium* are congeneric (Sri-indrasutdhi et al., 2010). *Canalisporium* has priority as it has fewer number of name changes and is an earlier name. Jones et al. (2016) and Réblová et al. (2016a) recommended the conservation of the generic name *Canalisporium* over *Ascothailandia*.

***Canalisporium caribense*** (Hol.-Jech. & Mercado) Nawawi & Kuthub. (1989), Figure 12
*Index Fungorum:* IF 125432; *FacesofFungi number*: FoF 05486

**FIGURE 12:**
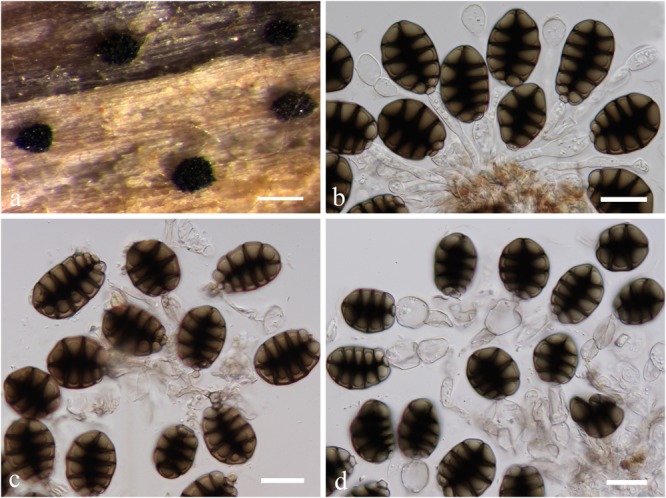
*Canalisporium caribense*(MFLU15**-**3581, holotype). **(a)** Substrate. **(b–d)** Sporodochia on wood. **(e,f)** Squash mount of a sporodochium. **(g)** Conidiophores. **(h,i)** Vesiculate conidiogenous cell. **(j)** Germinated conidium on nature substrate. **(k)** Germinated conidium on PDA medium. **(l–o)** Conidia. Scale bars: **(b)** 500 μm, **(c,d)** 50 μm, **(e,i–k)** 20 μm, **(f)** 30 μm, **(g,h)** 15 μm, **(l**–**o)** 10 μm.

**FIGURE 13:**
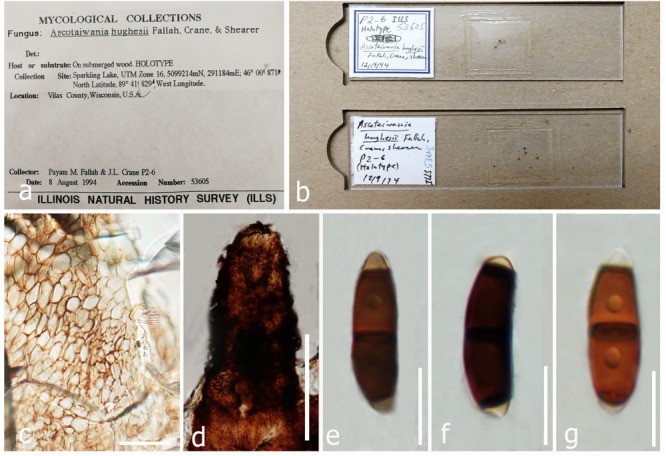
Sexual morph of *Helicoascotaiwania hughesii* (micro slides of holotype ILLS 53605). **(a,b)** Herbarium material. **(c)** Peridium. **(d)** Neck region. **(e–g)** Ascospores. Scale bars: **(d)** 100 μm, **(c)** 20 μm, **(e–g)** 10 μm.

*Description:* Please see Maharachchikumbura et al. (2016).

*Material examined*: Thailand, Chiang Rai Province, a stream flowing in Tham Luang Nang Non-Cave, on submerged wood, 25 November 2014, J. Yang (MFLU15**-**3581, holotype).

*Note*: *Canalisporium caribense*, the type species of this genus, is the latest name for *Berkleasmium caribense* (Holubová-Jechová and Sierra, 1984). Conidia of *Canalisporium* comprises septal canals, which is unique amongst taxa of hyphomycetes (Sri-indrasutdhi et al., 2010). *Canalisporium caribense*, however, has thick septa banded so that the canals are obscured by this heavy pigmentation, which is common in the freshwater environment (Nawawi and Kuthubutheen, 1989). The characteristic darkened pigmentation around the septa of the conidia is rather variable (Sri-indrasutdhi et al., 2010).

#### Key to *Canalisporium* Species

1. Conidiophore absent……………………***C. panamense***
1. Conidiophore present…………………………………**2**
2. Conidia with three small cells at the base and a single cell at the apex………………………………….. ***C. kenyense***
2. Conidia with a single cell at the base and one (rarely), two or multi cells at the apex………………………………**3**
3. Conidia with a column of longitudinal septa, scattered, pale olivaceous with clearly visible septa and canals, septa thin and not banded……………………………***C. pallidum***
3. Conidia with a single, double, or four to five columns of longitudinal septa, light brown to dark brown, septa often thick and brightly banded, canals obscured or not usually visible…………………………………………………**4**
4. Conidia comprising a column of longitudinal septa…………………………………………………**5**
4. Conidia comprising two or multi columns of longitudinal septa…………………………………………………**7**
5. Conidia with 3–6(–7) rows of transverse septa…………**6**
5. Conidia with 2–3(–4) rows of transverse septa.. ***C. exiguum***
6. Conidia 20–51 × 12–29 × (8–)10–16 μm, with 3–6(–7) rows of transverse septa………………………………**8**
6. Conidia with 25–50 × 13–19 × 6–10 μm, 4–5 rows of transverse septa………………………………………**7**
7. Conidia 25–34 × 13–19 × 6–10 μm, with 4–5 rows of transverse septa………………………………***C. nigrum***
7. Conidia 39–60 × 13–17.5 μm, with 4–5 rows of transverse septa…………………………………***C. thailandensis***
8. Conidia 24–51 × 15–29 × (8–)10–16 μm, with 3–6(–7) rows of transverse septa……………………***C. caribense***
8. Conidia 20–30 × 12–19 μm, with 3–5 rows of transverse septa……………………………………***C. dehongense***
8. Conidia 27–50 × 22–32 μm, 4–6 rows of transverse septa………………………………………***C. krabiense***
9. Conidia regularly with two columns of longitudinal septa…………………………………………………**10**
9. Conidia irregularly with 4–5 columns of longitudinal septa…………………………………………………**11**
10. Conidia with 2–4 rows, 1 cell at the apex, 22–35 × 15–23 × 10–10.5 μm, 2.5–5 wide………………***C. variabile***
10. Conidia with 2–4 rows, 1–4 cells at the apex, 25–33 × 20–28 × 7.5–11.5 μm, up to 25 μm long and 1.5–2 μm wide…………………………………***C. jinghongenses***
11. Conidia with 3–9 rows, 1–3 cells at the apex, 12.5–63 × 8–32 × 4–17 μm………………………………………**12**
11. Conidia with 5–8 rows, 1–5 cells at the apex, 32–58 × 25–38 × 10–13 μm……………………………***C. elegans***
11. Conidia with 4–6 rows, 3–4 cells at the apex, 27.5–37.5 × 24–27.5 × 17.5–22.5 μm…………***C. grenadoidia***
12. Conidia 25–63 × (16–)20–32 × 12–17 μm…***C. pulchrum***
12. Conidia 12.5–20 × 8–12 × 4–6 μm……***C. microsporum***

#### Genera Excluded From the Family

***Helicoon*** Morgan, J. Cincinnati. Soc. Nat. Hist. 15: 49 (1892)

**FIGURE 14:**
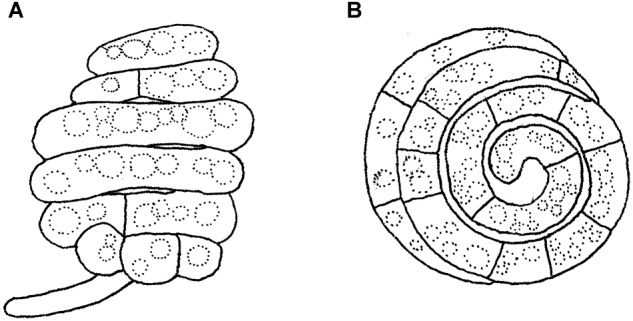
Asexual morph of *Helicoascotaiwania hughesii*. **(A,B)** Helicoid conidia. Scale bars: **(A,B)** = 20 μm (redrawn from Goos et al., 1986).

*Note*: Morgan (1892) introduced the genus *Helicoon*, an aero-aquatic hyphomycete characterized by the production of non-proliferating, cylindrical, barrel-shaped conidia (Figure 14). The conidia are borne on distinct conidiophores, which can be very short and inconspicuous (Fisher, 1977; Goos et al., 1986). According to MycoBank (Crous et al., 2004), NCBI^5^ (2018) and Wijayawardene et al. (2017) the genus *Helicoon* has 20 accepted species. Linder (1929) included seven of these in his monograph on helicosporous fungi. Nine species were listed by Moore (1955) in his key, and Abdullah (1980) described seven species from United Kingdom. Goos et al. (1986) included eight species in their key. The genus *Helicoon* was shown to be polyphyletic (Tsui and Berbee, 2006) although the sexual morph of *Helicoon* is presently unknown (Goos et al., 1986). *Ascotaiwania hughesii* was experimentally connected with a *Helicoon* asexual morph recognized as conspecific with *H. farinosum* (Fallah et al., 1999). *Helicoon farinosum* comprises hyaline, coiled, septate conidia that formed holoblastically on short denticles (Fallah et al., 1999) (Figure 14). In our phylogenetic analyses, *Ascotaiwania hughesii* along with its asexual morph *H. farinosum* grouped within Pleurotheciales and this is accepted by Réblová et al. (2012). Hence, we exclude *Helicoon farinosum* from Savoryellales. This is the only representative with helicosporous conidia in the Pleurotheciales and in subclass Savoryellomycetidae. *Helicoon sessile* (type species), was linked with *Orbilia* of the Orbiliales (Orbiliomycetes) (Pfister, 1997). The internal transcribed spacer sequence of *H. sessile* (U72605, Pfister, 1997) is identical to the sequence of *Sarocladium kiliense* in the Hypocreales (KP132606). Other phylogenetically related groups are *Tubeufiaceae* (*H. gigantisporum*), Pleosporales (*H. richonis*) or Dothideomycetes (*H. fuscosporum*). We could not obtain any type of material of *H. fuscosporum*.

***Monotosporella*** S. Hughes, Can. J. Bot. 36: 786 (1958)

*Note*: Hughes (1978) proposed *Monotosporella* for a hyphomycete with conidia produced by percurrent proliferation of conidiophores, isolated from wood in New Zealand and is typified by *Monotosporella setosa*. Subsequently, *Monotosporella* was synonymized under *Brachysporiella*; a genus comprising conidiophores that branch at the apex (Ellis, 1959). Later, Hughes (1958) suggested that *Monotosporella* should be different from *Brachysporiella*, because the lateral branches were not observed in any of the earlier introduced *Monotosporella* species. Seifert and Gams (2011) showed that *Monotosporella* is heterogenous while, Wijayawardene et al. (2012) listed *Monotosporella* under *Melanommataceae*. Presently, five species have been designated in *Monotosporella* (Index Fungorum, 2017). Sivichai et al. (1998) observed affinities between the asexual state of *Ascotaiwania* and *Monotosporella setosa*. Further, monotosporella-like asexual morphs have been reported for *A. mitriformis* and *A. sawadae* (Ranghoo and Hyde, 1998; Sivichai et al., 1998). The asexual morph *Ascotaiwania fusiformis* resembles *Monotosporella* species (Yang et al., 2016). Our phylogenetic analysis showed that *M. setosa* does not group in Savoryellaceae but within the order Pleurotheciales (please see Figure 19 in Tian et al., 2015, for the complete illustration of *M. setosa*). Hernández-Restrepo et al. (2013) concurred with Tian et al. (2015), and therefore we exclude *Monotosporella* from Savoryellaceae.

***Helicoascotaiwania*** Dayarathne, Maharachch. & K.D. Hyde **gen. nov.**
*Index Fungorum*: IF555625; *Facesoffungi number*: FoF 05487
*Etymology*: Name referring the similar morphologies to the genus *Ascotaiwania* and *Helicoon.*

*Saprobic* on decaying wood. **Sexual morph**: *Ascomata* perithecial, immersed, scattered, pyriform to obpyriform, black, straight or incline, with erumpent necks, membranous. Necks central, conical to cylindrical, rounded at apex, with a periphysate ostiolate. *Peridium* comprising several cell layers of brown, *textura angularis* in surface view, with 7–9(–12) compressed brown cells. *Paraphyses* 1.5–3 μm wide, branched, septate. *Asci* 8-spored, unitunicate, cylindrical, persistent, tapering to a long pedicel, with a J-apical ring. *Ascospores* uniseriate, fusiform, straight or curved, smooth-walled, 3-septate, central cells brown, polar cells hyaline, with a rounded apex and a wide base. **Asexual morph**: *Colonies* inconspicuous, white, powdery. *Conidiophores* micronematous, mononematous, subtle, rarely branched, hyaline, straight or flexuous, tapering upward, smooth. *Conidiogenous cells* monoblastic, integrated, terminal, determinate, denticulate. *Conidia* acrogenous, hyaline. *Conidial filament* coiled five to eight times in three planes to form a subglobose to ellipsoidal spore body (description based on our observations, Goos et al., 1986; Fallah et al., 1999).

*Type species*: *Helicoascotaiwania hughesii* (Fallah, J.L. Crane & Shearer) Dayarathne & K.D. Hyde

***Helicoascotaiwania hughesii*** (Fallah, J.L. Crane & Shearer) Dayarathne & K.D. Hyde **comb. nov**., Figure 13
*Index Fungorum*: IF555626*; Facesoffungi number*: FoF 05488
= ***Ascotaiwania hughesii*** Fallah, J.L. Crane & Shearer, Can. J. Bot. 77(1): 89 (1999)

*Saprobic* on decaying wood. **Sexual morph**: *Ascomata* 580–630 × 348–380 μm, immersed in decorticated wood, scattered, obpyriform, straight or tilted, with protruding beaks, membranous, black. *Necks* 200–225 × 100–140 μm, central, membranous, cylindrical to conical, broadly rounded at apex, periphysate, ostiolate. *Peridium* 40–45 μm wide, composed of several layers, brown, of *textura angularis* in surface view, composed of seven to nine compressed brown cells; cells 4.5–9 × 1.5–2 μm. *Paraphyses* 1.5–3 μm wide, branched, septate. *Asci* 263–347 × 9–10 μm, unitunicate, long-cylindrical, eight spored, persistent, tapering gradually to a long stalk, with an apical ring, 9–13.5 μm, J-, staining blue in aqueous cotton blue. *Ascospores* 18–30 × 6–8 μm, fusiform, uniseriate, straight or curved, smooth-walled, three septate, central cells brown, 8–10 μm long; end cells hyaline, rounded at apex, 4.5–5.5 μm wide at the base. **Asexual morph**: *Colonies* inconspicuous, white, powdery. *Conidiophores* micronematous, mononematous, inconspicuous, rarely branched, hyaline, straight or flexuous, tapering upward, smooth. *Conidiogenous cells* monoblastic, integrated, terminal, determinate, denticulate. *Conidia* acrogenous, hyaline. *Conidial filament* coiled five to eight times in three planes to form a subglobose to ellipsoidal spore body (modified description of Fallah et al., 1999 and based on our observations).

*Material examined*: United States, Wisconsin, Vilas Co., Sparkling Lake, UTM Zone 16, 291184 m E, 5099214 m N, 46°00’87”N, 89 41° ’83”W, on submerged wood, 8 August 1994, PMF P2-6, (micro slides of holotype ILLS 53605).

*Note*: *Helicoascotaiwania hughesii* (=*Ascotaiwania hughesii*) in reminiscent of *A. sawada* and *A. palmicola* in having 3-septate ascospores. It is distinguished from *A. sawada* by shorter ascospores with a cylindric-fusoid shape. The second septum of the ascospores is demarcated relatively closer to the apex in *A. hughesii* than that of *A. sawada*. The asci of *A. hughesii* are longer and narrower than those in *A. sawada* (Fallah et al., 1999). Ascomata of *Ascotaiwania hughesii* are larger and less rounded than in *A. palmicola* while the ascospores are longer and broader in *A. hughesii*. Ascospore appendages are present in *A. palmicola* but absent in *A. hughesii*. Fallah et al. (1999) reported that ascospores of *A. hughesii* germinated and formed white colonies and conidia were formed several days later, and all colonies were identified as belonging to the genus *Helicoon* (*Helicoon farinosum*). They also observed ascomata on the natural substratum, which discharged ascospores of *A. hughesii* among the conidia of *H. farinosum*. Further, sequences generated from both sexual morph and asexual morph strains did not clade with other *Ascotaiwania* members or within Savoryellales. They formed a well-supported clade within Pleurotheciaceae and the same result observed by Boonyuen et al. (2011); Yang et al. (2016), and Hernández-Restrepo et al. (2017). With the current phylogenetic placement and morphological differences of the asexual morph, we introduce a novel genus, *Helicoascotaiwania* to accommodate *A. hughesii* within Pleurotheciaceae.

## Discussion

Savoryellaceae is a fascinating family with taxa distributed in terrestrial and aquatic microhabitats (Boonyuen et al., 2011). There are several phylogenetic studies on this family, however, the family is not well resolved (Boonyuen et al., 2011). For instance, *Ascotaiwania* species was polyphyletic and the sexual and asexual morph links of certain species are not well-defined (Boonyuen et al., 2011). In our phylogram, the orders Conioscyphales, Fuscosporellales, Pleurotheciales, and Savoryellales clustered within the subclass Savoryellomycetidae, in a highly supported clade. In our phylogenetic analyses, Savoryellaceae formed a monophyletic group, with species belonging to *Ascotaiwania, Canalisporium, Neoascotaiwania, Savoryella, Bactrodesmium pallidum*, and *Triadelphia uniseptata*. Similar results were observed by Boonyuen et al. (2011) and Réblová et al. (2016b). *Canalisporium* and *Savoryella* are well-established monophyletic genera while *Ascotaiwania* was polyphyletic. Here, we resolved the polyphyly of *Ascotaiwania* by excluding *A. hughesii* (=*Helicoascotaiwania hughesii*) from the genus. We observed and re-described the type of *A. hughesii* (=*H. hughesii*) and provide a detailed description. Taxa previously referred to *Neoascotaiwania* comprise similar morphologies to species of *Ascotaiwania* and both taxa formed a monophyletic clade with high statistical support (100% ML, 1.00 PP), which indicates that *Neoascotaiwania* should be synonymized under *Ascotaiwania*. However, when comparing the available morphological data from different asexual morphs of *Ascotaiwania* it is clear that asexual morph characters are more informative in species recognition in *Ascotaiwania* when compared to their sexual morph, a view advanced by Hernández-Restrepo et al. (2017). Therefore, several name changes are undertaken in this study and summarized in Table 4. *Savoryella* species formed a well-supported (100% ML) monophyletic clade within Savoryellaceae. We introduced a novel species *S. yunnanensis* collected from China. *Savoryella yunnanensis* showed close phylogenetic affinities to *S. aquatica, S. fusiformis* and *S. verrucosa. Savoryella yunnanensis* morphologically resembles *S. lignicola*, however, the two species are phylogenetically distinct. Their bases pair differences of LSU 6 out of 826 (<1%), SSU 46 out of 1075 (4.27%), 12 out of 885 (1.3%) which are in adequate range to consider them as two distinct species as recommended by Jeewon and Hyde (2016). We observed the types of *S. aquatica, S. grandispora*, and *S. lignicola*, and report that the holotype of *S. aquatica* is in poor condition that has deprived us from observing any ascomata on the host substrate.

**Table 4:** Summary of name changes of this study.

Old name	Current name
*Ascotaiwania hughesii*	*Helicoascotaiwania hughesii*
*Helicoon farinosum*	*Helicoascotaiwania hughesii*
*Neoascotaiwania limnetica*	*Ascotaiwania limnetica*
*Neoascotaiwania terrestris*	*Ascotaiwania terrestris*

The divergence time estimations in the present study are congruent with former revisions (Beimforde et al., 2014; Pérez-Ortega et al., 2016; Samarakoon et al., 2016; Hyde et al., 2017). Our molecular clock analyses indicate that divergence times (crown age) of Sordariomycetes (325 Mya), is in agreement to those of the latest studies (317 Mya in Pérez-Ortega et al., 2016 and 309 Mya in Beimforde et al., 2014, 347 Mya in Samarakoon et al., 2016, 341 MYA in Hyde et al., 2017; Hongsanan et al., 2017). The divergence of Dothideomycetes crown group at 366 (400–492) Mya occurred before Sordariomycetes (325 Mya) (Liu et al., 2017). In our study, divergence times for most orders of Sordariomycetes are between 20 and 200 Mya, while divergence times for most families are between 10 and 100 Mya, which are consistent with those of Hyde et al. (2017). According to the available divergence time estimates of the marine ascomycete taxa, Koralionastetaceae and Lulworthiaceae might be the earliest with the stem age of 215 (145–283) Mya while the earliest marine lineage of Dothideomycete is reported for *Halottiaceae* with the stem age of 186 (133–135) (Liu et al., 2017).

Hongsanan et al. (2017) and Hyde et al. (2017) suggested that stem age of the subclass Sordariomycetes falls in the range of 250–289 Mya hence, Savoryellales is upgraded to subclass Savoryellomycetidae as its emergence goes back to early Mesozoic (201–252 Mya). This is supported by our study also and showed divergence time with stem age of 287 (232–343) for Sordariomycetes. The subclass Savoryellomycetidae includes four orders (Conioscyphales, Fuscosporellales, Pleurotheciales, and Savoryellales). The stem age of Pleurotheciales is around 143 Mya while it was reported as 139 Mya in Hongsanan et al. (2017); Conioscyphales, the sister clade of Pleurotheciales, evolved at the stem age of 180 Mya. Further, Fuscosporellales and Savoryellales have evolved at the stem ages of 249 Mya and 213 Mya, respectively. Therefore, order level status within Savoryellomycetidae range from 143–249 Mya, with families having crown ages of 104–182 Mya.

The occurrence of marine ascomycetes along with terrestrial or freshwater taxa provide evidence for the migration of ascomycetes from land to the marine environment (Spatafora et al., 1998; Vijaykrishna et al., 2006). Savoryellaceae is a good example for the above hypothesis comprising many terrestrial and freshwater genera as well as transition species such as *S. lignicola* (Pinruan et al., 2002; Vijaykrishna et al., 2006; Jones et al., 2009; Sakayaroj et al., 2011; Suetrong et al., 2015). The orders Koralionastetales, and Lulworthiales have co-evolved with a divergent age of 215 Mya which represents the most basal group. Additionally, the well-adapted marine taxa in the Halosphaeriaceae (order Microascales) and the marine order Torpedosporales showed divergence at about 171–241 Mya (Hongsanan et al., 2017). Vijaykrishna et al. (2006) documented that the *Halosphaeriaceae* evolved around 100 Mya, which is in agreement with the divergence time of 47–130 Mya in our study. However, molecular clock analyses provide various divergence time estimates for marine lineages and confirmed their evolution at different periods. The discovery of the extent new Caledonian species identified as *Monotosporella setosa* found developing on semi-solidified resin flows of *Agathis ovata* (Araucariaceae), is the first record of a *Monotosporella* species from modern resin substrates (Sadowski et al., 2012). Thus, the fossil age of this species could help to determine the exact phylogenetic placement of other *Monotosporella* species in the future. Most of the divergence time estimations have been conducted with mainly terrestrial representatives rather than those of marine origin and we recommend a thorough analysis of a wider range of marine fungal taxa.

## Author Contributions

AA-S, EJ, KH, KK, MD, RZ, SM, and VS planned the experiments. BD, WD, JY, MD, and WD conducted the experiments. AE, MD, and WD analyzed the data. BD, MD, SM, and EJ wrote the manuscript. All authors approved the manuscript.

## Conflict of Interest Statement

The authors declare that the research was conducted in the absence of any commercial or financial relationships that could be construed as a potential conflict of interest.

## Footnotes

1 http://sweetgum.nybg.org/ih/index.php

2 http://tree.bio.ed.ac.uk/software/figtree/

3 http://www.indexfungorum.org/names/NamesRecord.asp?RecordID=110142

4 http://www.indexfungorum.org/names/Names.asp

5 http://www.ncbi.nlm.nih.gov/nuccore (accessed August, 2018).
