# Supplementary material for: Phylogenetic Revision of Savoryellaceae and Evidence for Its Ranking as a Subclass
Source: Front Microbiol. 2019 May 7;10:840. doi: 10.3389/fmicb.2019.00840 (PMC6514050; doi:10.3389/fmicb.2019.00840)
Supplement: Supplementary file 1 [file Data_Sheet_1.doc]

**Supplementary tables**

**SUPPLEMENTARY TABLE 1.** GenBank accession numbers of isolates include in this study

| **Taxon** | **Strain no** | **LSU** | **SSU** | **RPB2** | **TEF1-α** |
| --- | --- | --- | --- | --- | --- |
| *Achroceratosphaeria potamia* | JF 08139 | GQ996538 | - | - | - |
| *Ambrosiella ferruginea* | CBS 460.82 | KM495316 | KR673890 | - | - |
| *Amphisphaeria umbrina* | AFTOL ID 1228 | FJ176863 | FJ176809 | FJ238348 | - |
| *Annulohypoxylon nitens* | MFLUCC 12-0823 | KJ934992 | - | KJ934994 | - |
| *Apiospora bambusa* | ICMP 6889 | DQ368630 | DQ368662 | DQ368649 | - |
| *Ascotaiwania fusiformis* | MFLUCC 15-0621 | KX550893 | - | KX576871 | - |
| *A. fusiformis* | MFLUCC 15-0625 | KX550894 | KX550898 | - | - |
| *A. lignicola* | NIL00005 | HQ446364 | HQ446284 | HQ446419 | HQ446307 |
| *A. limnetica* | CBS 126576 | KY853513 | KT278689 | - | - |
| *A. limnetica* | CBS 126792 | KY853514 | KT278690 | - | - |
| *A. mitriformis* | HKUCC3706 | AF132324 | - | - | - |
| *A. sawadae* | SS00051 | HQ446363 | HQ446283 | HQ446418 | HQ446306 |
| *A. terrestris* | CBS 142291 | NG058460 | NG061260 | - | - |
| *Ascothailandia grenadoidia* | SS03615 | GQ390267 | GQ390252 | HQ446420 | HQ446309 |
| *Asteridiella obesa* | VIC31239 | JX096809 | - | - | - |
| *Australiasca queenslandica* | BRIP 24607 | HM237324 | - | - | - |
| *Bactrodesmiastrum monilioides* | FMR 10756 | KF771879 | - | - | - |
| *B. monilioides* | FMR11337 | KF771877 | - | - | - |
| *B. obovatum* | FMR 6482 | FR870266 | - | - | - |
| *B. pyriforme* | FMR 10747 | FR870265 | - | - | - |
| *B. pyriforme* | FMR 11931 | HE646637 | - | - | - |
| *B. pallidum* | FMR 11345 | KY853485 | - | - | - |
| *Bartalinia robillardoides* | MFLUCC 12-2705 | KJ710438 | - | - | - |
| *Bombardia bombarda* | AFTOL ID 967 | DQ470970 | DQ471021 | DQ470923 | DQ471095 |
| *Buergenerula spartinae* | ATCC 22848 | DQ341492 | DQ341471 | - | JX134692 |
| *Canalisporium caribense* | SS03839 | GQ390268 | GQ390253 | HQ446421 |  |
| *C. elegans* | SS00877 | HQ446366 | HQ446286 | HQ446424 | HQ446311 |
| *C. exiguum* | SS00809 | GQ390281 | - | HQ446436 | - |
| *C. grenadoidia* | SS03615 | GQ390267 | GQ390252 | HQ446420 | HQ446309 |
| *C. jinghongensis* | SS03491 | GQ390272 | GQ390257 | HQ446426 | HQ446313 |
| *C. pallidum* | SS00498 | GQ390280 | GQ390265 | HQ446435 | HQ446322 |
| *C. pulchrum* | SS03773 | GQ390278 | GQ390263 | HQ446432 | HQ446319 |
| *C. krabiense* | MFLU 16-1888 | MH260283 | - | - | - |
| *C. thailandensis* | MFLU 16-1900 | MH260284 | - | - | - |
| *C. dehongense* | MFLU 18-1189 | MK051034 | MK051035 | - | - |
| *Carpoligna pleurothecii* | CBS 114211 | JQ429235 | JQ429249 | JQ429267 | - |
| *Ceratocystis adiposa* | CBS 600.74 | EU984304 | AY283562 | - | - |
| *C. fimbriata* | CBS 374.83 | - | AF221009 | DQ368641 | JX518297 |
| *Chaetosphaerella fusca* | GKML124N | FJ968967 | - | - | FJ969002 |
| *Chaetosphaerella phaeostroma* | SMH4257 | AY695264 | - | FJ968940 | FJ969004 |
| *Ciliochorella* sp. | MFLUCC 12-0310 | KF827445 | KF827446 | KF827479 | KF827477 |
| *Colletotrichum asianum* | LC0037 | JN940408 | JN940347 | - | JQ071902 |
| *C. fructicola* | LC0032 | JN940418 | JN940350 | - | - |
| *C. gloeosporioides* | LC0555 | JN940412 | JN940356 | - | - |
| *C. musae* | LC0962 | JN940415 | JN940353 | - | - |
| *Coniochaeta ligniaria* | C8 | AY198388 | - | - | - |
| *C. ostrea* | SMH2931 | DQ470959 | DQ471007 |  | DQ471078 |
| *Conioscypha japonica* | CBS 387.84 | AY484514 | JQ437438 | JQ429259 | - |
| *C. lignicola* | CBS 335.93 | AY484513 | JQ437439 | JQ429260 | - |
| *Conioscyphascus varius* | CBS 113653 | AY484512 | - | - |  |
| *Cordyceps agriota* | ARSEF 5692 | DQ518754 | DQ522540 | DQ522418 | DQ522322 |
| *C. aphodii* | ARSEF 5498 | DQ518755 | DQ522541 | DQ522419 | DQ522323 |
| *C. brunneipunctata* | OSC 128576 | DQ518756 | DQ522542 | DQ522420 | DQ522324 |
| *C. cf acicularis* | OSC 128580 | DQ518757 | DQ522543 | DQ522423 | DQ522326 |
| *C. irangiensis* | OSC 128578 | DQ518770 | DQ522556 | DQ522445 | - |
| *C. japonica* | OSC 110991 | DQ518761 | DQ522547 | DQ522428 | DQ522330 |
| *C.* sp. | NHJ 12581 | EF468831 | EF468973 | EF468930 | EF468775 |
| *C.* sp. | NHJ 12582 | EF468830 | EF468975 | EF468926 | EF468771 |
| *Cosmospora coccinea* | AR2741 | AY489734 | AY489702 | - | DQ471078 |
| *Crassochaeta nigrita* | SMH2931 | AY695266 | - | - | - |
| *Cryptosphaeria ligniota* | ATCC 46315 | KT425299 | - | KT425364 | - |
| *Cumulospora marina* | MF46 | GU252135 | GU252136 | - | - |
| *Diaporthe eres* | AR 3519 | AF362565 | - | - | KJ210547 |
| *Diatrype disciformis* | AFTOL-ID 927 | DQ470964 | DQ471012 | DQ470915 | DQ471085 |
| *Discosia brasiliensis* | MFLUCC 12-0431 | KF827437 | KF827441 | KF827474 | KF827466 |
| *Doratomyces stemonitis* | AFTOL-ID 1380 | DQ836907 | DQ836901 | - | - |
| *Elaphocordyceps capitata* | NBRC 100997 | JN941401 | JN941740 | - | - |
| *Exserticlava vasiformis* | TAMA 450 | AB753846 | - | - | - |
| *Fuscosporella pyriformis* | MFLUCC 16-0570 | KX550896 | KX550900 | KX576872 | - |
| *Gelasinospora tetrasperma* | CBS 178.33 | DQ470980 | DQ471032 | DQ470932 | - |
| *Gibellulopsis nigrescens* | DAOM 226890 | GU180648 | GU180613 | - | - |
| *Gliocephalotrichum bulbilium* | ATCC 22228 | AY489732 | AY489700 | - | AY489627 |
| *Gondwanamyces capensis* | AFTOL-ID 1907 | FJ176888 | FJ176834 | FJ238373 | - |
| *G. proteae* | AF221011 | AF221011 | - | - | - |
| *Graphium fimbriasporum* | CMW5605 | KM495388 | AY148171 | - | HM630590 |
| *G. penicillioides* | CBS 506.86 | AF027384 | - | - | - |
| *Graphostroma platystoma* | AFTOL-ID 1249 | DQ836906 | DQ836900 | DQ836893 | DQ836915 |
| *Halosphaeria appendiculata* | CBS 197.60 | U46885 | U46872 | - | FJ238390 |
| *Harknessia australiensis* | CPC 15029 | JQ706211 | - | - | - |
| *H. weresubiae* | CPC 17670 | JQ706244 | - | - | - |
| *Helicoascotaiwania hughesii* | DAOM 241947 | JQ429230 | - | - | - |
| *H. hughesii* | P2-6 | AY316357 | - | - | - |
| *Hydea pygmea* | NBRC 33069 | GU252133 | GU252134 | - | - |
| *Jugulospora rotula* | ATCC 38359 | AY346287 | - | AY780178 | - |
| *Koralionastes ellipticus* | JK5769 | EU863585 | EU863581 | - | - |
| *K. ellipticus* | JK5771 | EU863583 | EU863580 | - | - |
| *Kretzschmaria deusta* | CBS 163.93 | KT281896 | - | - | - |
| *Kylindria peruamazonensis* | CBS 838.91 | GU180638 | GU180609 | GU180656 | - |
| *Lasiosphaeria ovina* | SMH4605 | AY436413 | - | AY600284 | - |
| *Lecythophora luteoviridis* | CBS 206.38 | FR691987 | - | - | - |
| *Lecythothecium duriligni* | CBS 101317 | AF261071 | - | - | - |
| *Leotia lubrica* | AFTOL-ID 1 | NG027596 | AY544687 | DQ470876 | - |
| *Lignincola laevis* | AFTOL-ID 737 | U46890 | U46873 | *DQ836886* | - |
| *Lindra thalassiae* | AFTOL-ID 413 | DQ470947 | DQ470994 | DQ470897 | DQ471065 |
| *Lulworthia fucicola* | ATCC 64288 | AY878965 | AY879007 | - | - |
| *Magnaporthe poae* | M47 | JF414885 | JF414860 | - | JF710415 |
| *M. salvinii* | M21 | JF414887 | - | - | JF710406 |
| *Mazzantia napelli* | BPI748443 | AF408368 | DQ862051 | EU219345 | - |
| *Melanconis marginalis* | BPI748446 | AF408373 | DQ862053 | EU219301 | - |
| *M. stilbostoma* | CBS 109778 | AF408374 | NG013198 | EU219299 | - |
| *Melanospora tiffanii* | ATCC 15515 | AY015630 | AY015619 | AY015637 | - |
| *Meliola centellae* | VIC 31244 | JQ734545 | - | - | - |
| *Microascus longirostris* | CBS 196.61 | LN851043 |  |  | LM652566 |
| *M. trigonosporus* | AFTOL-ID 914 | DQ470958 | DQ471006 | DQ470908 | - |
| *Monilochaetes dimorphospora* | MUCL 40959 | HQ609480 | HQ609487 | - | - |
| *M. guadalcanalensis* | CBS 346.76 | GU180640 | - | - | - |
| *Monotosporella setosa* | HKUCC 3713 | AF132334 | - | - | - |
| *Mucispora obscuriseptata* | MFLUCC 15-0618 | KX550892 | KX550897 | KX576870 | - |
| *Neonectria ramulariae* | CBS 151.29 | HM042436 | HQ840414 | DQ789792 | JF735791 |
| *Neurospora crassa* | MUCL 19026 | AF286411 | X04971 | - | - |
| *Nimbospora effusa* | AFTOL-ID 761 | U46892 | U46877 | DQ836887 | - |
| *Ophiocordyceps acicularis* | OSC 110987 | EF468805 | EF468950 | - | EF468744 |
| *O. acicularis* | OSC 110988 | DQ518757 | DQ522543 | DQ522423 | - |
| *O. clavata* | NBRC 106961 | JN941414 | JN941727 | - | AB968586 |
| *O. clavata* | NBRC 106962 | JN941415 | JN941726 | EF468930 | AB968587 |
| *O. curculionum* | NHJ 12582 | KJ878885 | KJ878918 | - | EF468771 |
| *O. dipterigena* | OSC 151910 | KJ878886 | KJ878919 | - | - |
| *O. dipterigena* | OSC 151911 | KJ878887 | KJ878920 | - | KJ878966 |
| *O. elongata* | OSC 151912 | EF468808 | - | - | KJ878967 |
| *O. entomorrhiza* | KEW 53484 | EF468809 | EF468954 | EF468911 | EF468749 |
| *Ophiodiaporthe cyatheae* | HMH 2013 | JX570891 | JX570890 | JX570893 | KC465406 |
| *Parafuscosporella moniliformis* | MFLUCC 15-0626 | KX550895 | KX550899 | - | - |
| *Pestalotiopsis adusta* | CGMCC 3.9103 | JN940828 | JN940796 | - | - |
| *Petriella setifera* | AFTOL-ID 956 | DQ470969 | DQ471020 | - | DQ836911 |
| *Pisorisporium cymbiforme* | PRM 924379 | KM588903 | KM588900 | KM588906 | - |
| *Pleurotheciella rivularia* | CBS 125238 | JQ429232 | JQ429244 | JQ429263 | - |
| *Pleurothecium semifecundum* | CBS 131271 | JQ429240 | JQ429254 | JQ429270 | - |
| *Pseudomassaria_carolinensis_* | 9502 IFO | DQ810233 | DQ810262 | DQ810239 | - |
| *Psudoascotaiwania persoonii* | A57-14C | AY590295 | - | - | - |
| *P. persoonii* | A57-14C | AY094190 | - | - | - |
| *Remispora maritima* | BBH28309 | HQ111012 | HQ111002 | HQ111041 | - |
| *Robillarda sessilis* | CBS 101440 | KR873283 | - |  | KR873311 |
| *Savoryella aquatica* | SS03801 | HQ446372 | - | HQ446441 | HQ446326 |
| *S. fusiformis* | SS00783 | - | HQ446294 | HQ446443 | HQ446328 |
| *S. lignicola* | NF00204 | HQ446378 | HQ446300 | - | - |
| *S. lignicola* | NTOU791 | HQ446377 | HQ446299 | - | HQ446333 |
| *S. longispora* | SAT00322 | HQ446380 | HQ446302 | HQ446415 | HQ446336 |
| *S. paucispora* | SAT00866 | HQ446381 | HQ446303 | HQ446451 | HQ446337 |
| *S. verrucosa* | SS03331 | HQ446376 | HQ446298 | HQ446447 | SS03331 |
| *S. yunanensis* | MFLUCC 18-1395 | MK411422 | MK411423 | - | MK411424 |
| *Seimatosporium cornii* | MFLUCC 14-0467 | KR559739 | KR559741 | - | - |
| *Sordaria fimicola* | CBS 508.50 | AY681160 | - | - | - |
| *Stachylidium bicolor* | DAOM 226658 | GU180651 | GU180616 | - | - |
| *Thielaviopsis thielavioides* | CBS 130.39 | AF222480 | AF222518 | - | - |
| *Torpedospora ambispinosa* | BCC16003 | AY858949 | AY858940 | - | - |
| *T. mangrovei* | NBRC105264 | GU252149 | GU252150 | - | - |
| *T. radiata* | AFTOL-ID 751 | DQ470951 | DQ470999 | DQ470902 | DQ471070 |
| *Triadelphia uniseptata* | TA06NZ 142 | KT278718 |  |  |  |
| *Truncatella spartii* | It 1511 | KR092783 | KR092782 | - | - |
| *Umbrinosphaeria caesariata* | CBS 102664 | AF261069 | - | - | - |
| *Verticillium dahliae* | ATCC 16535 | AY489737 | AY489705 | - | AY489632 |
| *Vialaea minutella* | BRIP 56960 | KC181925 | - | - | - |
| *Xylaria bambusicola* | MFLUCC 11-0606 | KU863148 | - | KU940183 | - |
| *X. hypoxylon* | CBS 122620 | KM186301 | - | KY624231 | - |

Strains indicated in blue represents the new isolates obtained in this study.

**SUPPLEMENTARY TABLE 2** Different parameters for ML analysis

| **Analyses** | **Parameters** | **values** |
| --- | --- | --- |
| ML | Alignment patterns | 3116 |
|  | The proportion of undetermined characters or gaps. | 23.73% |
|  | Substitution model | GTR+I+G |
|  | Tree-length | 14.197816 |
|  | Estimated base frequencies | A = 0.248019 |
|  |  | C = 0.244647 |
|  |  | G = 0.286249 |
|  |  | T = 0.221085 |
|  | Substitution rates | AC = 1.099694 |
|  |  | AG = 2.578897 |
|  |  | AT = 1.066710 |
|  |  | CG = 1.223981 |
|  |  | CT = 5.482572 |
|  |  | GT = 1.000000 |
|  | Gamma distribution shape parameter | α = 0.367925 |
